# In *Arabidopsis thaliana* Substrate Recognition and Tissue- as Well as Plastid Type-Specific Expression Define the Roles of Distinct Small Subunits of Isopropylmalate Isomerase

**DOI:** 10.3389/fpls.2020.00808

**Published:** 2020-06-16

**Authors:** Kurt Lächler, Karen Clauss, Janet Imhof, Christoph Crocoll, Alexander Schulz, Barbara Ann Halkier, Stefan Binder

**Affiliations:** ^1^Institut für Molekulare Botanik, Fakultät für Naturwissenschaften, Universität Ulm, Ulm, Germany; ^2^DynaMo Center, Department of Plant and Environmental Sciences, Faculty of Science, University of Copenhagen, Frederiksberg, Denmark

**Keywords:** *Arabidopsis thaliana*, plastids, leucine biosynthesis, methionine-derived glucosinolates, isopropylmalate isomerase

## Abstract

In *Arabidopsis thaliana*, the heterodimeric isopropylmalate isomerase (IPMI) is composed of a single large (IPMI LSU1) and one of three different small subunits (IPMI SSU1 to 3). The function of IPMI is defined by the small subunits. IPMI SSU1 is required for Leu biosynthesis and has previously also been proposed to be involved in the first cycle of Met chain elongation, the first phase of the synthesis of Met-derived glucosinolates. IPMI SSU2 and IPMI SSU3 participate in the Met chain elongation pathway. Here, we investigate the role of the three IPMI SSUs through the analysis of the role of the substrate recognition region spanning five amino acids on the substrate specificity of IPMI SSU1. Furthermore, we analyze in detail the expression pattern of fluorophore-tagged IPMI SSUs throughout plant development. Our study shows that the substrate recognition region that differs between IPMI SSU1 and the other two IMPI SSUs determines the substrate preference of IPMI. Expression of IPMI SSU1 is spatially separated from the expression of IPMI SSU2 and IPMI SSU3, and IPMI SSU1 is found in small plastids, whereas IMPI SSU2 and SSU3 are found in chloroplasts. Our data show a distinct role for IMPI SSU1 in Leu biosynthesis and for IMPI SSU2 and SSU3 in the Met chain elongation pathway.

## Introduction

Amino acids serve as building blocks for proteins but also have other important functions. In plants for instance, they are initial substrates for the biosynthesis of specialized metabolites such as glucosinolates in the family of the Brassicaceae. According to the parent amino acid, glucosinolates are grouped into three major categories. Aromatic glucosinolates derive from Phe or Tyr whereas those synthesized from Trp represent the indole glucosinolates. The highly diversified aliphatic glucosinolates originate from Ala, from the branched-chain amino acids (Val, Leu, and Ile) or from Met. The compounds derived from Met represent the most important subgroup of aliphatic glucosinolates in terms of quantity and diversity (Wittstock and Halkier, 2002; Halkier and Gershenzon, 2006).

**FIGURE 1:**
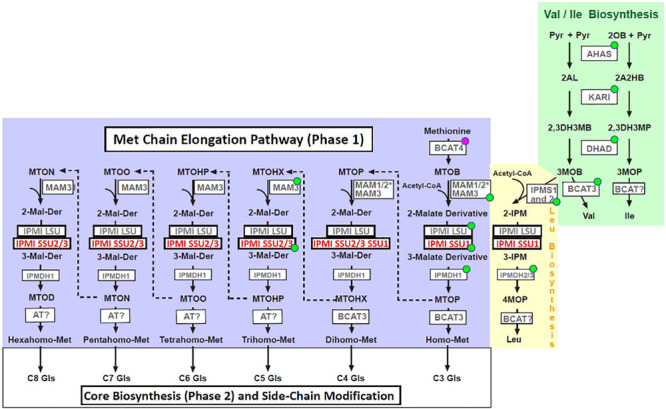
Metabolites and enzymes of branched-chain amino acid biosynthesis (Val and Ile: green box, Leu: yellow box) and methionine chain elongation pathway (blue box). Abbreviations of metabolites: Pyr, pyruvate; 2OB, 2-oxobutanoate; 2AL, 2-acetolactate; 2A2HB, 2-aceto-2-hydroxybutyrate; 2,3DH3MB, 2,3-dihydroxy-3-methylbutanoate; 2,3DH3MP, 2,3-dihydroxy-3-methylpentanoate; 3MOB, 3-methyl-2-oxobutanoate; 3MOP, 3-methyl-2-oxopentanoate; 4MOP, 4-methyl-2-oxopentanoate; 2-IPM, 2-isopropylmalate; 3-IPM, 3-isopropylmalate; MTOB, methylthiooxobutanoate; MTOP, methylthiooxopentanoate; MTOHX, methylthiooxohexanoate; MTOHP, methylthiooxoheptanoate; MTOO, methylthiooxooctanoate; MTON, methylthiooxononanoate; MTOD, methylthiooxodecanoate, 2-Malate-Derivative, 3-Malate-Derivative; Gls, glucosinolate. Abbreviations of enzymes: AHAS, acetohydroxyacid synthase; KARI, ketolacid reductoisomerase; DHAD, dihydroxyacid dehydratase; BCAT, branched-chain aminotransferase; IPMS, isopropylmalate synthase; MAM, methylthioalkylmalate synthase; IPMI LSU, isopropylmalate isomerase large subunit; IPMI SSU, isopropylmalate isomerase small subunit; IPMDH, isopropylmalate dehydrogenase; AT, aminotransferase. Enzymes marked with a green dot localize to plastids according to SUBA4 (http://suba.live/, Hooper et al., 2017). BCAT4 is located in the cytosol. *The presence or absence of MAM1 or MAM2 is ecotype-dependent. Both enzymes are involved in early phases of Met chain elongation leading to predominantly C4 (MAM1, for instance in Col) or C3 (MAM2, for instance in Ws) glucosinolates (Kroymann et al., 2003).

Met-derived glucosinolates are synthesized in three phases (Figure 1). The first phase, the Met chain elongation pathway, is initiated by a transamination of Met to 4-methylthio-2-oxobutanoate (MTOB), a reaction catalyzed by the cytosolic branched chain aminotransferase 4 (BCAT4), which acts at the interphase between primary and specialized metabolism (Schuster et al., 2006). This reaction product is imported into plastids (Gigolashvili et al., 2009; Sawada et al., 2009b), where it is elongated by three-step cycles including condensation with acetyl-CoA (catalyzed by methylthioalkylmalate synthases, MAM), followed by isomerization (isopropylmalate isomerase, IPMI) and oxidative decarboxylation (isopropylmalate dehydrogenase, IPMDH). Each cycle results in a 2-oxo acid elongated by a single methylene group [i.e., 5-methylthio-2-oxopentanoate (MTOP) after a single cycle], which can now re-enter the pathway and undergo one or several additional rounds of elongation (condensation, isomerization and oxidative decarboxylation, up to eight methylene groups) (Grubb and Abel, 2006), or it is transaminated to a Met derivative. The latter metabolites are exported from the plastids to enter glucosinolate core biosynthesis along the endoplasmatic reticulum. In the last stage, various side chain modifications further increase the diversity of the Met-originating glucosinolates (Wittstock and Halkier, 2002; Halkier and Gershenzon, 2006).

Branched chain amino acids are synthesized in plastids. In a first part, two parallel pathways starting from two molecules of pyruvate or pyruvate and 2-oxobutanoate, 3-methyl-2-oxobutanoate (3MOB) and 3-methyl-2-oxopentanoate (3MOP) are formed in reactions catalyzed by three enzymes [acetohydroxyacid synthase (AHAS), ketolacid reductoisomerase (KARI) and dihydroxyacid dehydratase (DHAD)]. While 3MOP is transaminated to Ile [by a branched-chain aminotransferase (BCAT)], 3MOB can be transaminated to Val or it can be chain-elongated to 4-methyl-2-oxobutanoate (4MOP) in a reaction cascade identical to Met chain elongation demonstrating the close relationship between these pathways (condensation, isomerization and oxidative decarboxylation, Figure 1; Kroymann et al., 2001; Schuster et al., 2006; Binder, 2010). The close relationship is underpinned by homologous enzymes active in these reaction cascades. This is most obvious for proteins such as the large subunit of isopropylmalate isomerase (IPMI LSU1), which functions both in Leu biosynthesis and in the Met chain elongation pathway (Knill et al., 2009; Sawada et al., 2009a; He et al., 2010).

Leu and Met-derived glucosinolates are representatives of the primary and specialized metabolism, respectively, with completely different biological roles. Given the close relationship of the reaction cascades of both pathways involving homologous enzymes, the formation of Leu and the chain elongation of Met have to be kept apart. The biosynthesis of glucosinolates derived from Met occurs in cells associated with vascular tissues (Reintanz et al., 2001; Chen et al., 2003; Grubb et al., 2004; Tantikanjana et al., 2004; Schuster et al., 2006; Gigolashvili et al., 2007, 2008; He et al., 2010; Li et al., 2011; Imhof et al., 2014; Nintemann et al., 2018) and these specialized metabolites are stored in S-cells (Sulfur-rich cells) situated in close vicinity to the phloem (Koroleva et al., 2000, 2010). In contrast, very little is known where and when Leu biosynthetic proteins are expressed and therefore it is completely unknown how the biosynthesis of this amino acid is kept apart from the Met chain elongation pathway.

To address this issue, we now investigated the expression of the three small subunits of isopropylmalate isomerase (IPMI SSU1 to 3) in *Arabidopsis thaliana* (*Arabidopsis*). Using translational fusions to different fluorescent proteins, we examined the spatial expression of the small subunit 1 of isopropylmalate isomerase (IPMI SSU1, representative for Leu biosynthesis) and of the small subunits 2 and 3 (IPMI SSU2 and IPMI SSU3, representative for Met elongation) on the tissue, cell and organelle level. These investigations revealed a spatial separation of IPMI SSU1 on the one hand from IPMI SSU2 and IPMI SSU3 on the other hand. Apart from the differential accumulation of the small subunits in terms of space, distinct substrate specificities, as analyzed by converting three amino acid identities in the substrate recognition region of IPMI SSU1 to identities present in IPMI SSU3, contribute to the specialized function of isopropylmalate isomerases in *Arabidopsis*.

## Materials and Methods

### Plant Lines and Cultivation

*Arabidopsis* plants were grown on standard soil containing 20% Vermiculite grain size 2–3 mm (Isola-Mineralwolle-Werke GmbH, Germany) and 1.5 g/l Osmocote Exact Mini fertilizer (Scotts GmbH, Germany). Plants were cultivated in a growth chamber under the following conditions: 16 h light (100–150 μmol/m^2^s)/8 h dark at 21°C. Dual reporter lines were established by crossing characterized lines IPMI SSU1:RFP, IPMI SSU2:ECFP and IPMI SSU3:GFP according to a standard protocol (Weigel and Glazebrook, 2002). Plant lines IPMI SSU1:RFP, *ipmi ssu2/ipmi ssu3, ipmi ssu2/ipmi ssu3* + pSSU3:SSU1 and *ipmi ssu2/ipmi ssu3* + SSU3 had been described in a previous study (Imhof et al., 2014). For selection of transformants, seeds were germinated on MS medium containing 0.5% (w/v) sucrose and 50 μg/ml hygromycin.

### Nucleic Acids Methods

To determine the insertion sites of the T-DNAs in the IPMI SSU1:RFP, IPMI SSU2:ECFP and IPMI SSU3:GFP reporter lines, thermal asymmetric interlaced (TAIL) PCR was performed on total DNA isolated following a previously reported protocol (Edwards et al., 1991). TAIL PCRs followed a previously established procedure (Liu and Whittier, 1994). Shortly, three T-DNA-specific oligonucleotides (TR-1 to TR-3) and the Degenerated-Primer.1 were used to amplify sequences across the T-DNA left border and adjacent genomic regions in the three nested amplification reactions. In the first PCR, total DNA was used as template while the following two nested PCRs were done on amplification products generated in the previous reactions. A typical PCR program used in these experiments is given in Supplementary Method S1. Control reactions were done applying the same procedure but starting out from total DNA obtained from non-transformed wild-type plants. In comparison to the control reactions, specific products were selected and sequenced to determine the T-DNA insertion site. The localizations of the T-DNAs of the IPMI SSU1:RFP, IPMI SSU2:ECFP and IPMI SSU3:GFP reporter lines are given in Supplementary Table S1.

### Construct Cloning

Cloning of the construct for the expression of the *IPMI SSU1* open reading frame under the control of the *IPMI SSU3* promoter (pSSU3:SSU1) was described in a previous report (Imhof et al., 2014). An overlap extension PCR strategy was applied to exchange amino acid identities from FLTLV (IPMI SSU1) to YGTLI (IPMI SSU3) in the *IPMI SSU1* coding region. To this end, two PCR products overlapping by 19 nucleotides were amplified with primer pairs SSU3comp.H/SSU3-1FOE.R2 and SSU1FOE.H/SSU1comp.R. These products were purified applying the GenElute Gel Extraction Kit (Sigma-Aldrich) and used as template in subsequent amplification reaction. To this end, both template PCR products were hybridized at 60°C for 5 s followed by five PCR cycles in the absence of additional primers resulting in an extension of the overlapping DNA strands. Then primers ssu3comp. H and SSU1comp. R were added and the gene with altered substrate recognition region (IPMI pSSU3:SSU1/SSU3srr) was amplified in 35 cycles. The product was finally cloned into the *Asc*I/*Pac*I sites in pMDC99 (Curtis and Grossniklaus, 2003) and the correct cloning and codon exchange was confirmed by DNA sequencing. The constructs were then used to transform *ipmi ssu2-1/ipmi ssu3-1* double knockout plants using *Agrobacterium* strain GV2260 following the floral dip procedure (Clough and Bent, 1998). The resulting line was designated *ipmi ssu2-1/ipmi ssu3-1*/ + pSSU3:SSU1/SSU3srr.

To investigate the tissue-, cell and organelle-specific localization of the three IPMI SSUs the complete reading frames and promoters were fused in frame to the red fluorescent protein *Entacmaea quadricolor* epFP611 (RFP), to the enhanced cyan fluorescent protein (ECFP) or to the soluble modified green fluorescent protein (smGFP) (Davis and Vierstra, 1998; Zacharias et al., 2002; Forner and Binder, 2007). The construct for the expression of the IPMI SSU1:RFP fusion protein had been described previously (Imhof et al., 2014) and the corresponding line is referred to as IPMI SSU1:RFP. To clone the reporter fusion construct for IPMI SSU2 and ECFP, the *IPMI SSU2* gene (−981 to +768) was amplified with oligonucleotides SSU2-ECFP.H(*Pac*I) and SSU2-ECFP.R(*Sgs*I)2 on *Arabidopsis* wild-type DNA and cloned into the corresponding sites in pMDC99. The *ECFP* reading frame was amplified with primers pSAT6.H(*Bam*HI) and pSAT6.H(*Pst*I) (Tzfira et al., 2005) and inserted into the *Bam*HI/*Pst*I restriction sites downstream of the *IPMI SSU2* gene. To follow IPMI SSU3 expression the corresponding gene (−1012 to +759) was amplified on total *Arabidopsis* DNA with oligonucleotides SSU3-GFP. H and SSU3-GFP.R, digested with *Bam*HI and *Sma*I and cloned into plasmid psmGFP4 upstream of the *GFP* reading frame (Davis and Vierstra, 1998). The fused IPMI SSU3:GFP reading frame including the IPMI SSU3 promoter was then excised from this plasmid with restriction enzymes *Bam*HI and *Eco*RI and cloned into the corresponding sites in pMDC99 (IPMI SSU3:GFP). All constructs were introduced into the *ipmi ssu2/ipmi ssu3* double knockout plants as mentioned above. After selection and inspection of different transformants by PCR, the insertion sites of the T-DNA were determined by TAIL PCR as described above and homozygous lines were established. To simultaneously follow expression of two different IPMI SSUs in single plants, the lines were crossed to generate IPMI SSU1:RFP/IPMI SSU2:ECFP, IPMI SSU1:RFP/IPMI SSU3:GFP and IPMI SSU2:ECFP/IPMI SSU3:GFP dual reporter lines. In addition, we cloned an alternative construct for the expression of fusion protein consisting of IPMI SSU1 and the ECFP, which is named IPMI SSU1:ECFP. To this end the *IPMI SSU1* gene (−431 to +753) was amplified with primer pair SSU1-ECFP.H(*Pac*I)/SSU1-ECFP.R(*Sgs*I) and the corresponding fragment was cloned into the *Pac*I/*Sgs*I sites in the above described construct IPMI SSU2:ECFP, where it replaces the *IPMI SSU2* gene. In this case, T-DNA insertion site was not determined in the transgenic plants.

For the analysis of cell-specific expression of BCAT4 an approximately 2.0 kb promoter fragment was amplified using primer pair BCAT4.Prom.hin/BCAT4.Prom.rueck. The fragment was digested with *Bam*HI and *Asc*I (sites introduced with primers) and cloned into the corresponding sites upstream of the reading frame of triple GFP containing a C-terminal nuclear localization signal (3xGFP-NLS derived from vector pJF667), that has been inserted before via *Asc*I and *Pac*I sites in pMDC99. The BCAT4 promoter:3XGFP-NLS (pBCAT4:3xGFP-NLS) construct was used to transform *Arabidopsis* as outlined in previous sections.

### Microscopy

Fluorescence microscopy was done with a Leica DM5500 equipped with a Leica DFC3000 G digital camera with filter sets: RFP: RFP excitation 545/30 nm, beam splitter 570 nm, emission 610/75 nm; GFP: GFP excitation 480/40 nm, beam splitter 505 nm, emission 527/30 nm, ECFP excitation 436/20 nm, beam splitter 455 nm, emission 480/40 nm and chlorophyll autofluorescence: excitation 470/40, beamsplitter 500, emission 525/50.

In addition, images were captured with a confocal microscope Leica SP5X equipped with a HCX PL APO lambda blue 20.0 × 0.70 IMM UV objective. ECFP was excited at 458 nm. Emission was collected at 468–520 nm for ECFP and 650–695 nm for chloroplast autofluorescence. GFP was excited at 488 nm. Emission was collected at 500–530 nm for GFP and 650–695 nm for chloroplast autofluorescence. Images were analyzed using the LAS AF and LAS X software (Leica). Except for images shown in Figures 5C,D, 6B and Supplementary Figure S3, all images were taken with wide field microscopy. Depending on the number of cell layers in the distinct cross sections, individual organelles and their shape cannot be resolved clearly in individual wide field images.

### Miscellaneous Methods

Glucosinolates were analyzed following a protocol established previously (Brown et al., 2003). Twenty mg seeds were ground using a ball mill and the homogenized material was rehydrated in 1 ml 80% (v/v) methanol. After the addition of 50 μl of Sinalbin as internal standard, samples were incubated for 5 min at room temperature and then centrifuged at 13,000 rpm for 10 min. Eight hundred μl of the supernatants were recovered and loaded on equilibrated DEAE-Sephadex A 25 columns. Columns were washed with 500 μl 80% (v/v) methanol, two times with 1 ml double distilled water and 500 μl 0.02M MES buffer pH 5.2. Glucosinolates bound to the column matrix were then treated with 30 μl arylsulfatase for desulfation (sulfatase from Helix pomatia, Sigma-Aldrich) over night and finally eluted with 0.5 ml double distilled water. The eluted desulfoglucosinolates were separated using high performance liquid chromatography (Agilent 1100 HPLC system, Agilent Technologies) on a reversed phase C-18 column (Nucleodur Sphinx RP, 250 × 4.6 mm, 5 μm, Macherey-Nagel, Germany) with a water (A)-acetonitrile (B) gradient (0–1 min, 1.5% B; 1–6 min, 1.5–5% B; 6–8 min, 5–7% B; 8–18 min, 7–21% B; 18–23 min, 21–29% B; 23–23.1 min, 29–100% B; 23.1–24 min 100% B and 24.1–28 min 1.5% B; flow 1.0 ml min-1). Metabolites were detected with a photodiode array detector and peaks were integrated at 229 nm. Desulfoglucosinolates were identified by comparison of retention time and UV spectra to those of purified standards previously extracted from *Arabidopsis thaliana*.

For each line, eight seed pools each derived from seven or eight plants were analyzed and considered for calculation. Only for line *ipmi ssu2-1/ipmi ssu3-1* complemented with IPMI SSU3 (*ipmi ssu2-1/ipmi ssu3-1* + SSU3), six pools were considered for evaluation, since two lines were clear outliers. Sequencing was obtained commercially (4baselab^1^, LGC Genomics^2^. Alignments were done using the clustalW alignment tool^3^. Oligonucleotide sequences are given in Supplementary Table S2.

## Results

### Amino Acid Identities in the Small Subunits of the IPMI Influence the Substrate Range of the Heterodimeric Enzyme

**FIGURE 2:**
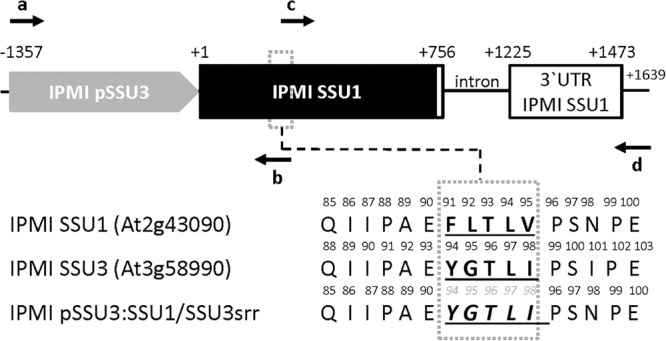
Schema of the construct used to test the importance of distinct amino acids in the substrate recognition region (SRR) in the small subunits of IPMI. The *IPMI SSU1* reading frame (black box) including its native 3′ untranslated region (3′ UTR), white box) is expressed under the control of the *IPMI SSU3* promoter (gray box). In the SRR (dotted box) three amino acids originally present in IPMI SSU1 were substituted by the corresponding amino acid present in IPMI SSU3. Original amino acid sequences and the resulting new sequence (IPMI pSSU3:SSU1/SSU3srr) are given in the lower part of the figure. Oligonucleotides used for the generation of the construct and the amino acid replacement are indicated as horizontal arrows. Theses primers are: a, ssu3comp.H; b, SSU3/1FOE.R2, c, SSU1FOE. H and d, SSU1comp.R. Oligonucleotide sequences are given in Supplementary Table S2. Numbering is given with respect to the translation start codons. Schema not drawn to scale.

It was previously suggested that the three small subunits of isopropylmalate isomerase have different roles in Leu and/or glucosinolate metabolism in *Arabidopsis* (He et al., 2010; Imhof et al., 2014). To investigate whether the distinct functions are related to different substrate specificities influenced by the small subunits of the enzyme, we exchanged amino acid identities in a region potentially influencing the interaction with different substrates. Studies in prokaryotes identified a substrate recognition region (SRR) spanning five amino acids in the small subunit of the heterodimeric IPMI (Yasutake et al., 2004; Lee et al., 2012). A comparison of the three SSUs from *Arabidopsis* revealed differing amino acid identities within the SRR, which might be responsible for the distinct function of these proteins (Supplementary Figure S1). In a previously used construct, which allows the expression of the *IPMI SSU1* reading frame under the *IPMI SSU3* promoter (Table 1, *ipmi ssu2-1/ipmi ssu3-1* + pSSU3:SSU1), we replaced several amino acids in the substrate recognition region in the *IPMI SSU1* reading frame by amino acid residues present in this region in *IPMI SSU3* (Figure 2, IPMI pSSU3:SSU1/SSU3srr). This construct was introduced into a *ipmi ssu2-1/ipmi ssu3-1* double knockout mutant (Table 1, *ipmi ssu2-1/ipmi ssu3-1* + pSSU3:SSU1/SSU3srr). The double knockout line is unable to synthesize detectable amounts of C6, C7 and C8 aliphatic glucosinolates (Table 1, *ssu2-1/ipmi ssu3-1*) (Imhof et al., 2014).

**TABLE 1:** Glucosinolate content in seeds of different lines.

	**Glucosinolate Content in Seeds (μmol/g)**					
	
**Gls**	**Col−0**	**Ws**	***ipmi ssu2-1/ipmi ssu3-1***	***ipmi ssu2-1/ipmi ssu3-1* + pSSU3:SSU1**	***ipmi ssu2-1/ipmi ssu3-1* + pSSU3:SSU1/SSU3srr**	***ipmi ssu2-1/ipmi ssu3-1* + SSU3**
3BZO	3.42015	10.270.42	23.070.67	12.161.66	17.642.21	5.470.94
3OHP	0.500.10	3.210.26	11.430.28	5.620.58	4.260.99	1.530.37
3MTP	0.030.03	20.883.82	4.570.28	0.270.10	1.361.12	0.080.02
3MSOP	0.100.03	5.152.12	1.840.23	0.160.07	0.570.46	0.110.09
4BZO	14.171.09	0.070.02	2.950.11	10.810.98	7.691.85	8.820.94
4MTB	16.441.31	n.d.	9.970.49	25.964.01	23.275.15	32.748.18
4OHB	4.390.29	n.d.	2.740.13	12.600.74	5.260.56	5.030.31
4MSOB	2.670.60	0.570.14	3.590.36	2.340.40	2.440.51	4.841.64
5MSOP	0.720.23	0.290.21	0.490.20	0.340.05	0.470.15	1.010.31
5MTP	1.900.29	n.d.	n.d.	0.410.04	0.630.33	2.140.38
6MSOH	0.460.14	0.190.10	n.d.	n.d.	0.480.18	0.660.11
7MSOH	2.010.48	0.880.30	n.d.	n.d.	0.970.26	2.790.17
7MTH	5.760.87	3.110.34	n.d.	n.d.	3.730.58	6.480.56
8MSOO	8.951.08	9.901.72	n.d.	n.d.	0.570.26	9.480.90
8MTO	6.850.77	14.391.93	n.d.	n.d.	0.600.15	4.810.47
I3M	1.160.12	0.310.03	0.390.03	0.340.03	0.500.08	0.640.16
total	69.534.35	69.246.56	61.031.42	71.017.18	70.438.89	86.6411.53

*3BZO, 3-benzoyloxypropylglucosinolate; 3OHP, 3-hydroxypropylglucosinolate; 3MTP, 3-methylthiopropylglucosinolate; 3MSOP, 3-methylsulfinylpropylglucosinolate; 4BZO, 4-benzoyloxybutylglucosinolate; 4MTB, 4-methylthiobutylglucosinolate; 4OHB, hydroxybutylglucosinolate; 4MSOB, 4-methylsulfinylbutylglucosinolate; 5MSOP, 5-methylsulfinylpentylglucosinolate; 5MTP, 5-methylthiopentylglucosinolate; 6MSOH 6-methylsulfinylhexylglucosinolate; 7MSOH, 7-methylsulfinylheptylglucosinolate; 7MTH, 7-methylthioheptylglucosinolate; 8MTO, 8-methylthiooctylglucosinolate; 8MSOO, 8-methylsulfinyloctylglucosinolate and I3M, indol-3-ylmethylglucosinolate. Number of samples measured for the different lines is given in section “Materials and Methods.” srr, substrate recognition region; p, promoter.*

Transgenic plants were selected by their resistance to hygromycin and the presence of the construct was confirmed by PCR. Glucosinolate profiling of seed samples obtained from individual T1 transformants revealed that the exchange of the amino acid identities in the SRR allowed the synthesis of 6-methylsulfinylhexylglucosinolate (6MSOH), 7-methylsulfinylheptylglucosinolate (7MSOH), 8-methylthiooctylglucosinolate (8MTO) and 8-methylsulfinyloctylglucosinolate (8MSOO), albeit they do not reach the levels seen upon the introduction of an intact IPMI SSU3 gene into the double knockout mutant. In contrast, these C6, C7 and C8 aliphatic glucosinolates were absent from the *ipmi ssu2*/*ipmi ssu3* double knockout mutant and in mutant plants containing the unchanged construct for the expression of the native *IPMI SSU1* open reading frame under the control of the *IPMI SSU3* promoter (Table 1). These results demonstrated that the synthesis of these long chain glucosinolate species correlates with the exchange of the amino acid identities in the SRR. Thus, this region contributes to the substrate specificity of heterodimeric IPMI in *Arabidopsis*. The clear difference in glucosinolate composition between the *ipmi ssu2-1/ipmi ssu3-1* double knockout mutant and this line containing the construct IPMI pSSU3:SSU1/SSU3srr (*ipmi ssu2-1/ipmi ssu3-1* + pSSU3:SSU1/SSU3srr) indicated that the mutated small subunit can functionally interact with the large IPMI subunit (Table 1).

### Reporter Lines Used to Analyze Spatial Expression of Small Subunits of IPMI

To investigate whether the different roles of the three small subunits of the isopropylmalate isomerase come along with differential spatial expression *in planta*, we established reporter lines expressing full length IPMI SSU proteins N-terminally fused to different fluorescent proteins in the *ipmi ssu2-1/ipmi ssu3-1* double knockout line (IPMI SSU1:RFP, IPMI SSU1:ECFP, IPMI SSU2:ECFP, and IPMI SSU3:GFP). These fusion proteins were expressed under the native promoters, which allowed a tissue-, cell-, and cell compartment-specific visualization of these proteins. For direct comparison of the different expression patterns we also established dual reporter lines, expressing two different fusion proteins (IPMI SSU1:RFP/IPMI SSU2:ECFP, IPMI SSU1:RFP/IPMI SSU3:GFP, and IPMI SSU2:ECFP/IPMI SSU3:GFP). We also generated a line expressing a nucleus-targeted triple green fluorescent protein controlled by the promoter of *BCAT4* (pBCAT4:3XGFP-NLS), a gene involved in Met chain elongation (Schuster et al., 2006). An overview of the various constructs is given in Supplementary Figure S2. After the establishment of the reporter lines, various tissue samples were taken from plants grown on MS medium or soil under long-day conditions.

### Inspection of Different Tissues From Young Seedlings

**FIGURE 3:**
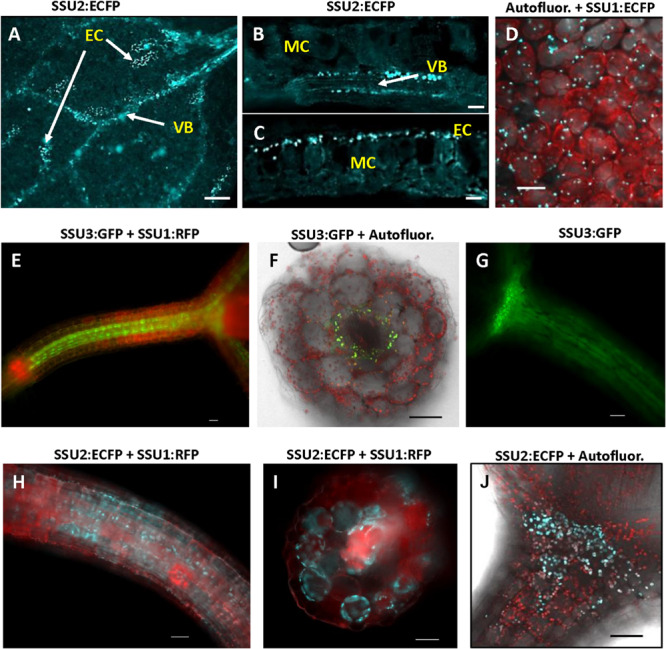
Localization of the small subunits of IPMI in different parts of young seedlings. **(A–D)** cotyledons, **(E–J)** hypocotyls. Whole mount **(A)** and cross sections of cotyledons **(B,C)**. IPMI SSU2:ECFP (cyan) is seen in the cells along the vascular bundles (VB) in cotyledons **(A,B)** and in isolated epidermis cells [EC in **(A,C)**]. MC indicates mesophyll cells. **(D)** View onto a leaf indicating that the fluorescence of IPMI SSU1:ECFP (cyan) is mainly detected in plastids with a longitudinal diameter of approximately 4–5 μm in the epidermis. **(E–J)** IPMI SSU1-3 expression in the hypocotyl. **(D,F,H,J)** Autofluor., chlorophyll autofluorescence (red). Scale bars correspond to 20 μm **(B,C)**, 50 μm **(A,D–J)**.

An inspection of cotyledons from 4 to 7-day-old seedlings identified expression of IPMI subunits 1 and 2 in these organs. Consistent with previous analyses applying promoter:GUS constructs, IPMI SSU2 expression was found in tissues along the vascular bundles (Imhof et al., 2014). In addition, IPMI SSU2 also localized to isolated epidermal cells distributed over the cotyledon lamina (Figure 3A). Cross sections clearly confirmed the localization of IPMI SSU2 in epidermal cells and the vascular bundles (Figures 3B,C). IPMI SSU1:ECFP accumulated in approximately roundish, slightly elongated organelles similar to mesophyll-plastids but much smaller with a longitudinal diameter of about 4–5 μm within epidermal cells covering large parts of the lamina (Figure 3D). Usually no autofluorescence was seen in these organelles, but in rare instances, typical chlorophyll autofluorescence was detected, which confirmed that these organelles are indeed plastids (Supplementary Figure S3).

The longitudinal microscopic inspection of the hypocotyls of the different reporter lines revealed the presence of IPMI SSU2 and IPMI SSU3 in plastids of cells, which show a slight difference with respect to their location along the central vascular bundle. While IPMI SSU3 seems to be located in close vicinity to the central vasculature (Figure 3E), IPMI SSU2 was also seen in further peripherally located cells (Figure 3H) with some spatial overlap of the tissues expressing these proteins. Expression of both proteins extends up to the cotyledoneous node (Figures 3G,J). Cross sections through the hypocotyl confirmed that the IPMI SSU3:GFP protein localized to plastids in parenchyma cells surrounding the central vasculature, whereas IPMI SSU2 was also found in further peripheral cells (Figures 3F,I). These plastids containing IPMI SSU2 and IPMI SSU3 have sizes comparable to chloroplasts (Figures 3F,J). The localization of IPMI SSU1 differed substantially from IPMI SSU2 and IPMI SSU3. The IPMI SSU1/IPMI SSU2 and IPMI SSU1/IPMI SSU3 dual reporter lines demonstrated that IPMI SSU1 is preferentially located in peripheral cells of the hypocotyls (Figures 3E,H,I).

**FIGURE 4:**
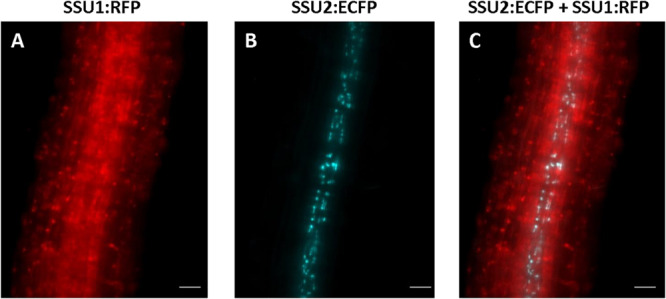
Expression of IPMI SSU1 **(A)** and IPMI SSU2 **(B)** in roots of 6-day-old seedlings. An overlay of images is given in **(C)**. Scale bar is 20 μm **(A–C)**.

In seedling roots, IPMI SSU1 occurred in the epidermis including root hairs and throughout the cortex whereas IPMI SSU2 was found in cells along the vasculature (Figures 4A–C and Supplementary Figure S4). The IPMI SSU1:RFP/IPMI SSU2:ECFP and IPMI SSU1:RFP/IPMI SSU3:GFP dual reporter lines showed an expression of IPMI SSU1 in root parenchyma, clearly different from the localization of the other subunits involved in the Met chain elongation pathway (Figures 4A,B). This expression pattern observed for IPMI SSU1 is consistent with previous observations (Imhof et al., 2014). In roots, the analyzed proteins are generally found in small plastids with a size as described above.

### Distribution of the Different Small Subunits of IPMI in Leaves

**FIGURE 5:**
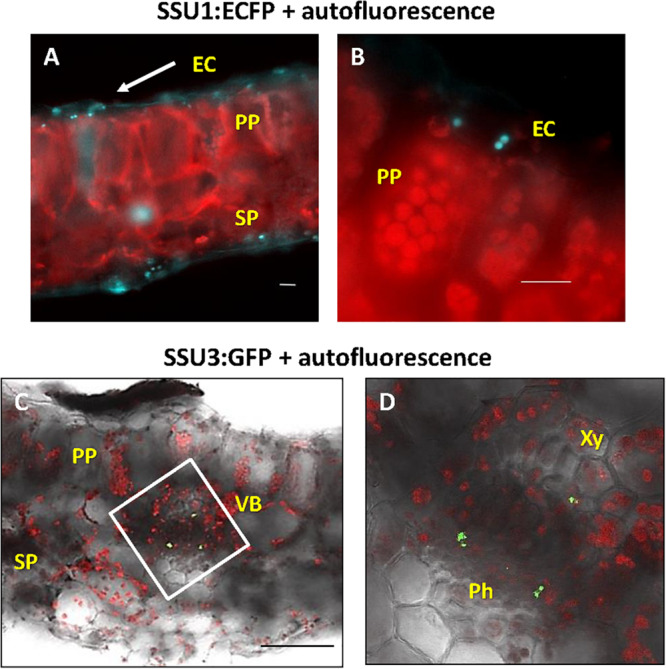
Localization of the small IPMI subunits in leaves. **(A,B)** IPMI SSU1:ECFP-positive plastids (cyan) locate to the leaf epidermis. **(B)** the epidermal cells (EC) contain small plastids with longitudinal diameter of 4–5 μm. In comparison, chloroplasts (red autofluorescence) of mesophyll cells [palisade parenchyma (PP) and sponge parenchyma (SP)] are 7–8 μm in size. **(C,D)** Cross section through a leaf vascular bundle (VB) revealed IPMI SSU3:GFP in cells located proximal to both phloem (Ph) and xylem (Xy), respectively. Scale bars: **(A,B)** 20 μm, **(C)** 100 μm. **(D)** is a magnification of the white boxed part of the image shown in **(C)**.

In rosette leaves, ECFP-tagged IPMI SSU1 was found in plastids of both, the upper and lower epidermis (Figures 5A,B). In these cells, the fusion protein marked organelles close to the cell wall toward the mesophyll. These plastids were clearly smaller than the chloroplasts in mesophyll cells (Figure 5B). IPMI SSU3:GFP marked plastids in cells in the phloem and in cells close to the xylem along the vascular bundles as seen in cross sections (Figures 5C,D). These IPMI SSU3 containing organelles have sizes very similar to chloroplasts (highlighted by the chlorophyll autofluorescence) in the immediate vicinity.

### Spatial Differentiation of IPMI SSU Expression in Roots

**FIGURE 6:**
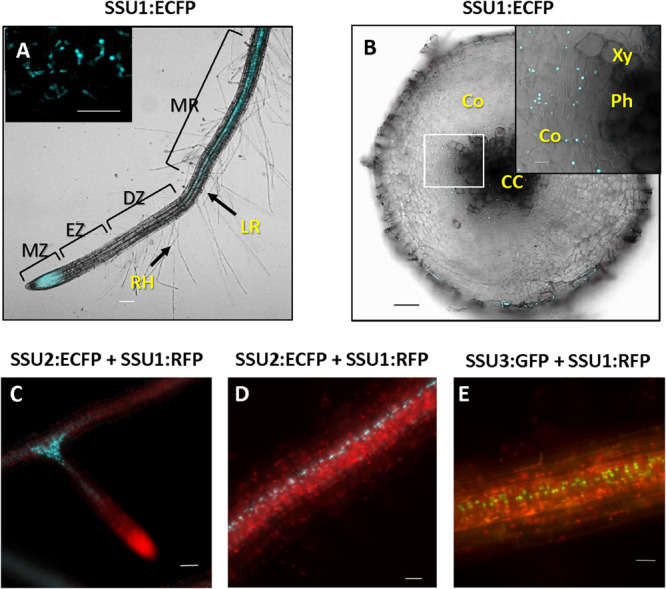
**(A)** Whole mount root with IPMI SSU1:ECFP fluorescence visible in the meristematic zone (MZ) and along the stele of mature root region (MR). IPMI SSU1 is not observed in the elongation (EZ) and differentiation zones (DZ). Root hairs (RH) and an outgrowing lateral root (LR) are indicated. **(B)** In an older part of a mature root, IPMI SSU1 is also detectable in the cortex (Co) surrounding the central cylinder (CC) but not in the vascular tissue itself [xylem (Xy) and phloem (Ph)]. IPMI SSU1 containing plastids form stromules [inset in **(A)**]. **(C–E)** IPMI SSU2 and IPMI SSU3 are seen in plastids along the central cylinder, with a strong accumulation of these organelles at branches (here shown for IPMI SSU2). In contrast, IPMI SSU1 is seen in plastids throughout the cortex and in the root tip **(C)**. Scale bars: inset in **(A)** 10 μm, **(B)** 100 and 50 μm, **(C–E)** 20 μm.

The three small subunits of IPMI are also expressed in roots of adult plants. IPMI SSU1 containing plastids were prevalent in the root tip and seem to be less evident in the elongation and differentiation zone (Figures 6A,C). Organelles with IPMI SSU1 were also found in the cortex of the mature root enclosing the stele (Figures 6B,D,E). These plastids formed stromules (Figure 6A, inset). In contrast, IPMI SSU2 and IPMI SSU3 were seen in cells closely associated with the stele (Figures 6D,E).

### The Enzymes of Met Chain Elongation IPMI SSU2, IPMI SSU3 and BCAT4 Are Expressed in Parenchyma Cells Associated With the Phloem or the Xylem in Flowering Stalks

**FIGURE 7:**
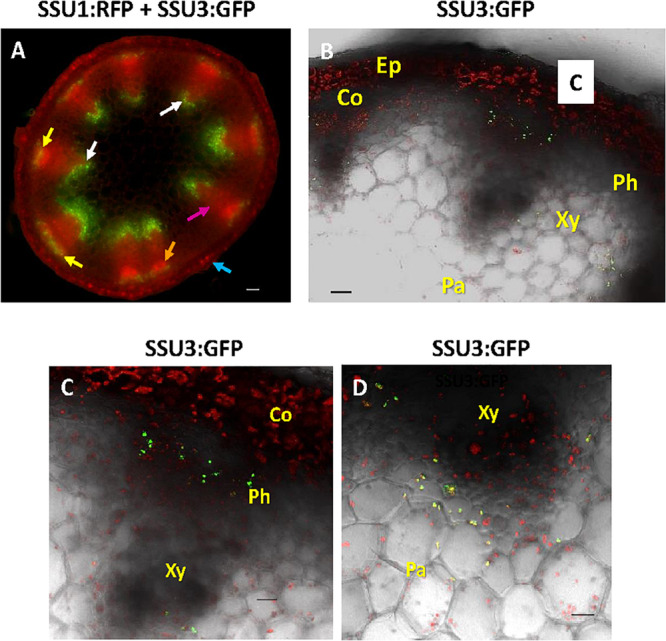
Cross section through a flowering stalk. **(A)** In a dual reporter line, IPMI SSU3:GFP expression is seen in cells associated with the phloem (indicated by yellow arrows) but also in parenchyma cells proximal to the xylem (white arrows, with some background autofluorescence of lignin in the xylem), whereas IPMI SSU1 labeled with RFP lights up in the epidermis (blue arrow) and also in phloem-associated cells (orange arrow), although a clear resolution of the organelles is not possible in this image. Background red autofluorescence is exemplarily indicated by a purple arrow. **(B–D)** Magnified cross section shows IPMI SSU3:GFP co-expression in cells associated with the phloem (Ph) and the xylem (Xy). Other abbreviations: Co, cortex; Ep, epidermis. **(C)** IPMI SSU3:GFP in cells in close proximity to the phloem. **(D)** IPMI SSU3:GFP in cells located proximal to the xylem. Scale bars: 50 μm **(A)**, 20 μm **(B–D)**.

Expression studies of genes involved in glucosinolate biosynthesis and its regulation revealed promoter activities in or along the vascular tissues. In this context, it has been speculated that the promoter activity might co-localize with glucosinolate storing S-cells (Koroleva et al., 2000; Schuster et al., 2006). To investigate the exact cellular and subcellular localization of the IPMI SSUs, we inspected cross sections through the flowering stalk. In an IPMI SSU3/IPMI SSU1 dual reporter line, these studies provided evidence that IPMI SSU3 was expressed in two populations of cells, associated with either the phloem or the xylem (Figure 7A, yellow and white arrows). The cross section indicated IPMI SSU1 in the epidermis (Figure 7A, blue arrow). The image of this section also suggested that this protein might also be present in phloem-associated cells (Figure 7A, orange arrow). This localization was confirmed by a longitudinal section of a flowering stalk of IPMI SSU2/IPMI SSU1 dual reporter plant. The IPMI SSU1-containing plastids were small and found to be clearly different from the chloroplasts containing IPMI SSU2 (Supplementary Figure S5A). Higher magnification images localized IPMI SSU3 to a few cells located at the periphery of the phloem (Figures 7B,C) and in cells located proximal to the xylem (Figures 7B,D), here found in plastids with very similar sizes to the chloroplasts in the close vicinity (Figure 7D). To investigate whether the observed pattern is valid only for isopropylmalate isomerase subunits 2 and 3 or whether other proteins of the Met chain elongation pathway are also expressed in these parenchymatic cells, we analyzed BCAT4 promoter activity with a 3XGFP-NLS reporter gene (Supplementary Figures S5B,C). Longitudinal sections showed that BCAT4 is also expressed in cells in distal parts of the phloem and like IPMI SSU3, also in cells proximal to the xylem (Supplementary Figures S5B,C). Along the phloem, 3XGFP-NLS fluorescence was detected in nuclei of relatively short cells that exhibited a morphology completely different from the extremely long S-cells (Supplementary Figure S5B) (Koroleva et al., 2000, 2010). In addition, S-cells are located further distal to those cells expressing BCAT4, which are located very close to the phloem (Supplementary Figure S5C). Interestingly, nuclei indicating BCAT4 expression in the xylem parenchyma were often found to be elongated whereas those demonstrating BCAT4 expression in the cells linked to the phloem were predominantly of globular shape (Supplementary Figure S5C).

### Occurrence of the Different Small Subunits in Generative Tissues

**FIGURE 8:**
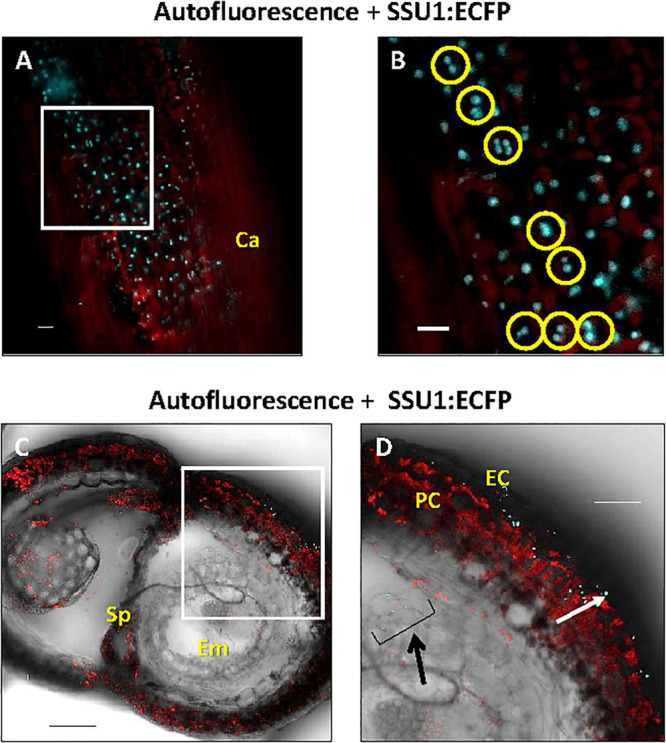
Cellular and subcellular localization of the IPMI SSU1 protein in a premature silique. **(A)** An overlay of the fluorescence of IPMI SSU1:ECFP (cyan) and the chlorophyll autofluorescence (red) indicates that IPMI SSU1 is located in small sensory plastid-like organelles within cell layers above the chloroplast-containing cells (Ca, carpel). **(B)** A magnification of a part of the image shown in (**A,** white box) highlights the presence of IPMI SSU1 in dividing plastids (yellow circles). **(C,D)** In a cross section, IPMI SSU1:ECFP fluorescence (cyan) can be seen in small plastids (with a longitudinal diameter of approximately 4–5 μm) in epidermal cells (EC). In contrast, pericarp cells (PC) contain many chloroplasts as indicated by the chlorophyll fluorescence (red). In addition, IPMI SSU1:ECFP fluorescence is visible in outer cell layers in the developing seed (indicated by a black arrow). Sp, septum; Em, embryo. Scale bars: **(A)** 100 μm, **(B)** 10 μm and **(C,D)** 50 μm.

Finally, we examined the *in planta* distribution of the three IPMI SSUs in generative tissues. In carpels, IPMI SSU1 was found in relatively small plastids that did not exhibit detectable autofluorescence (Figure 8A). These plastids seemed to be located in the epidermis above a chloroplast-containing cell layer. Interestingly many of these plastids were observed in pairs indicating dividing stages (Figure 8B). Expression of IPMI SSU1 was neither detected in sepals, petals nor stamen, while IPMI SSU2 expression was associated with the vasculature in sepals and petals. A cross section through a maturing silique revealed the expression of IPMI SSU1 in the outermost cell layer consistent with the localization of this protein in the epidermis of the carpel (Figure 8C). The plastids labeled by IPMI SSU1:ECFP are clearly smaller than chloroplasts (Figure 8D). In pre-mature seeds, IPMI SSU1 was found in small plastids in a cell layer close to the surface very similar to what has been observed in leaves or carpels (Figure 8D). No autofluorescence was detected in the IPMI SSU1:ECFP-positive plastids and again a relatively large number of these organelles seemed to be in a dividing stage.

**FIGURE 9:**
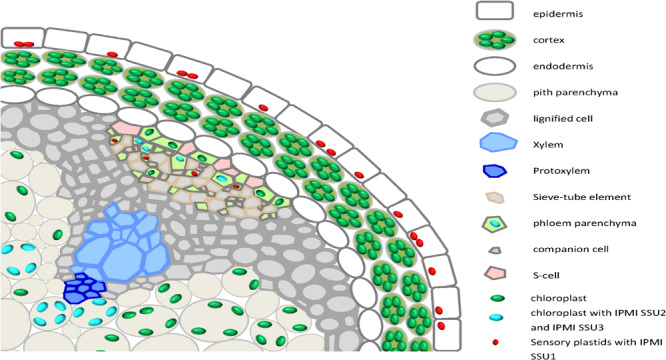
A cartoon summarizing the expression of the different IPMI SSU proteins in a cross section through the flowering stalk.

Collectively these data demonstrate an almost strict correlation of the different functions of the IPMI small subunits with distinct localizations in different plastids, cells and tissues. The localization patterns are summarized in a cartoon showing a cross section through the flowering stalk (Figure 9).

## Discussion

### Sequences in the IPMI SSU Contribute to the Substrate Specificity of the Heterodimeric Enzyme

Isopropylmalate isomerase, which catalyzes the second step in Leu biosynthesis, occurs in two forms (Singh, 1999; Binder, 2010). The single subunit or monomeric enzyme is found in fungi whereas the heterodimeric form is found in bacteria and archaea as well as in plants (Knill et al., 2009; Manikandan et al., 2011; Lee et al., 2012). The heterodimer is built up of a large subunit, which contains an iron-sulfur cluster and a small subunit. Studies in prokaryotes showed that the active site of the enzyme is formed at the interface of both components (Lee et al., 2012). In the small subunit, two regions are critical for substrate binding. The GSSRE substrate binding motif, which is highly conserved in all small IPMI polypeptides and in related aconitases, and the substrate recognition region (SRR) located in a flexible loop of these proteins (Supplementary Figure S1; Frishman and Hentze, 1996; Gruer et al., 1997; Jeyakanthan et al., 2010; Lee et al., 2012). In *Arabidopsis* and rice, the GSSRE motif as well as N- and C-terminally flanking amino acids are perfectly conserved, consistent with the conserved function of this motif in substrate binding but at the same time excluding a role of this motif in substrate differentiation. Thus, the SRR determines the substrate preference of the heterodimeric enzyme consistent with previous studies of IPMI and homoaconitase (HACN) in *Methanocaldococcus jannaschii* (Jeyakanthan et al., 2010). Within the SRR, at least three amino acids differ between the three small subunits of *Arabidopsis* (Figure 2 and Supplementary Figure S1). In line with the apparent function of the single small IPMI subunit from rice in Leu biosynthesis, the SRR region from this protein is almost identical to the *Arabidopsis* IPMI SSU1 that functions in Leu metabolism (Supplementary Figure S1; Imhof et al., 2014). The exchange of three amino acids in IPMI SSU1 to adopt the SRR sequence from IPMI SSU3 and the expression of this modified reading frame under the *IPMI SSU3* promoter in the *ipmi ssu2-1/ipmi ssu3-1* double mutant resulted in an altered glucosinolate composition. Notably, the otherwise undetectable C6, C7, and C8 glucosinolate species accumulated after the amino acid substitutions in the SRR, albeit these compounds did not reach the levels that were measured when the native IPMI SSU3 was introduced into the *ipmi ssu2-1/ipmi ssu3-1* double knockout mutant. This suggests that additional amino acids at other positions influence the substrate specificity of the IPMI, but the lower levels might be also linked to differences in expression since the transgenes in the distinct lines originate from different transformation events. The differences observed in the composition in the C3 and C4 glucosinolates could be linked to a segregation of the methylthioalkylmalate synthase 1 (MAM1, Col) and MAM2 (Ws) genes in the *ipmi ssu2-1/ipmi ssu3-1* double knockout mutant. Ecotypes containing MAM1 synthesize mainly C4 short-chain glucosinolates while in those accessions with MAM2 C3 glucosinolates are dominant (Table 1 and Figure 1; Kroymann et al., 2001, 2003). The generation of long-chain glucosinolates is linked to the activity of MAM3 found in all ecotypes (Figure 1; Kroymann et al., 2003; Textor et al., 2007). Thus, the accumulation of the long-chain glucosinolates (C6 to C8) in lines *ipmi ssu2-1/ipmi ssu3-1*/+pSSU3:SSU1/SSU3srr and *ipmi ssu2-1/ipmi ssu3-1*/+pSSU3:SSU1is independent from the genetic background of the recipient line *ipmi ssu2-1/ipmi ssu3-1*, in which no C6 to C8 glucosinolate species were detected (Table 1; Imhof et al., 2014), but is solely linked to the introduced transgenes.

The fact that the introduced construct for the expression of the SRR-modified IPMI SSU1 (line *ipmi ssu2-1/ipmi ssu3-1* + pSSU3:SSU1/SSU3srr) altered the glucosinolate composition in comparison to the acceptor line, i.e., the *ipmi ssu2-1/ipmi ssu3-1* double mutant, demonstrates that a functional dimerization occurred and that the introduced amino acid exchanges did not interfere with binding between this protein and the IPMI large subunit. This scenario is further supported by observations made by the analysis of bacterial and archaeal IPMI and the closely related homoaconitase (HACN), which is like IPMI composed of a small and a large subunit. Changes in the SRR region of HACN in *M. jannaschii* had no negative influence on heterodimer formation. Even the exchange of 28 amino acids at the N-terminus of HACN with the sequence from the small subunit of IPMI did not interfere with higher order structure formation (Jeyakanthan et al., 2010). Furthermore, the small IPMI subunit of the archaeon *Pyrococcus horikoshii* can functionally replace the small HACN subunit when introduced into *Termus thermophilus*. This observation demonstrates that a functional dimer is formed between the large subunit HACN and the small subunit of IPMI although this small subunits shares only 43.0% identical and 62.8% similar amino acids with the small HACN subunit (Lombo et al., 2004).

Since the sequences in the SRR differ between all three IPMI subunits in *Arabidopsis* there is the potential to build IPMI enzymes with three different substrate preferences. In case of IPMI SSU1 (FLTLV), this corresponds with an expression pattern completely different from IPMI SSU2 and IPMI SSU3. In hypocotyls, there seems to be also a subtle difference between the expression pattern of IPMI subunits involved in Met chain elongation. The amino acid sequences in the SRRs are different between of IPMI SSU2 (AACTF) and IPMI SSU3 (YGTLI). While the latter contains two aliphatic amino acid at position 4 and 5 similar to IPMI SSU1, amino acid with aliphatic characteristics are present in positions 1 and 2 of IPMI SSU2. However, previous studies did not reveal clear differences in the glucosinolate quantity or composition between corresponding single mutants and wild-type lines. Likewise, amino acid profiling did not give clear hints toward the function of these proteins in the metabolism of theses primary metabolites (Knill et al., 2009; He et al., 2010). In summary, our data demonstrate that substrate specificities of the IPMI SSU1-containing heterodimer and most likely of all IPMI heterodimers in *Arabidopsis* are determined by amino acid identities in the SRRs of the small subunit of IPMI and that this can contribute to functional specification of the holoenzymes. However, further detailed studies are required to define the exact substrate specificities of the individual IPMI SSUs in *Arabidopsis*.

Similar to IPMI, a functional specification was also observed for the isopropylmalate dehydrogenases. Here two enzymes are primarily involved in Leu biosynthesis (IPMDH2 and 3) whereas IPMDH1 catalyzes the oxidative decarboxylation step in the Met chain elongation pathway (He et al., 2009, 2011a). An amino acid substitution (Phe to Leu) in the active site severely reduces the activity of IPMDH1 toward 3-(2′-methylthio)-ethylmalate, an intermediate of Met chain elongation and enhances the conversion of 3-isopropylmalate while the reverse substitution (Leu to Phe) in IPMDH2 has the reciprocal effect. Similar to the small subunits of IPMI, the functional specification of these enzymes seems to be also influenced by spatiotemporal expression of the different IPMDH genes (He et al., 2011b).

### Expression of Small Subunits of IPMI in Young Seedlings

All small IPMI subunits are expressed in young seedlings. IPMI SSU1 was found in small plastids in the epidermis similar to what was also seen in true leaves (Figures 3A,C, 5A,B). In most images, no natural fluorescence was detected in these small organelles with a longitudinal diameter of approximately 4 to 5 μm, but their detailed inspection revealed a weak autofluorescence linked to the presence of chlorophyll (Supplementary Figure S3). This is consistent with the recent detection of a set of proteins required for photosynthesis in so called sensory plastids located in vascular parenchyma cells. The sensory plastids, probably identical with what was known as leucoplasts in textbooks (Hudák, 2005), were also found in leaf epidermal cells and are morphologically very similar or even identical to the IPMI containing plastids seen in this analysis (Virdi et al., 2016; Beltran et al., 2018). Apart from cotyledons IPMI SSU1 was also found in parenchymatic cells surrounding the central vasculature in hypocotyls and roots (Figures 3E,H,I).

IPMI SSU2 and IPMI SSU3 were found along the vascular tissue in all parts of the seedlings. This is consistent with previous histochemical analyses of IPMI pSSU2:GUS and IPMI pSSU3:GUS reporter lines (He et al., 2010; Imhof et al., 2014). But now our analysis revealed a slightly different expression pattern of both proteins in hypocotyls, which might contribute to functional specification (Figures 3E,H). But as stated above, the knockout or knockdown of one or two genes in various plant lines did not result in any changes in glucosinolate quantity or composition, possibly be due to the measurement of total green tissue from adult plants, which does not detect differences between distinct tissues (Knill et al., 2009; He et al., 2010; Imhof et al., 2014).

A very peculiar expression pattern of IPMI SSU2 was seen in cotyledons. Here we found this protein in individual cells in the epidermis beside the usual association with vascular bundles (Figures 3A–C). Also expression of TGG1, a glucosinolate degrading myrosinase, has been found in individual epidermis cells of cotyledons, but the pattern seems to be completely different from that of IPMI SSU2 suggesting no co-localization of this protein and the myrosinase (Husebye et al., 2002). Strikingly, the epidermal expression of IPMI SSU2 was found to be restricted to the cotyledon stage since we did not observe a similar pattern in rosette leaves as it has been seen for flavin monoxygenase FMO_GS–OX1_ (Li et al., 2011).

### IPMI SSU1 Expression Occurs Predominantly in Small Plastids of Epidermal Cell Layers in Green Tissues of Adult Plants

In textbooks and reviews, amino acid biosynthesis is usually described to occur in so-called leucoplasts with no details given on the localization of these organelles in distinct cell types or tissues (Hudák, 2005). Our studies of adult plants confirmed that IPMI SSU1 fusion proteins accumulate in small plastids distinguishable from chloroplasts (Figures 3D, 5B). In rosette leaves, the major part of the above-ground tissue of *Arabidopsis*, IPMI SSU1-containing plastids were exclusively detected in epidermis cells. This localization in the epidermis is consistent with two other studies (Svozil et al., 2015; Beltran et al., 2018). As mentioned above, the size of these organelles and their localization in epidermis cells are consistent with so-called sensory plastids (Virdi et al., 2016; Beltran et al., 2018). A proteomic analysis of FACS separated sensory plastids from vascular parenchyma and mesophyll chloroplasts confirmed the presence of IPMI SSU1 in the proteome of sensory plastids and the absence of this protein from mesophyll chloroplasts. This applies also for other proteins of the leucine biosynthetic pathway, strongly suggesting that the complete biosynthetic pathway is present in this organelle type (Beltran et al., 2018). In addition, the localization of IPMI SSU1 both in epidermal cells and in cells associated with phloem is perfectly reflected by a targeted proteome analysis of leaf mesophyll, of epidermal and of vascular tissue of *Arabidopsis* leaves (Svozil et al., 2015). This study found IPMI SSU1 predominantly in the epidermis and to a lower extent also in vascular tissue, but not in the mesophyll, in contrast to the exclusive localization of BCAT4, IPMI SSU2 and IPMI SSU3 in the vasculature. Although sensory plastids from vascular tissue and from epidermal cells share a set of identical proteins, it is presently unclear to which extent the proteomes of these organelles from both tissues match each other (Beltran et al., 2018).

Sensory plastids contain the MutS HOMOLOG1 (MSH1) suggested to be a stress response signaling protein, highlighting the role of these organelles in tissue-specific signaling of stress response in plants. Under abiotic stress conditions, MSH1 steady state mRNA is strongly reduced, whereas downregulation or knockout of the plastid-located MSH1 proteins signal a stress state to the mutant plant, which displays enhanced tolerance to abiotic stress (MacKenzie and Kundariya, 2019). Consistent with this observation, the proteome composition of these sensory plastids reflects the function in triggering of stress and developmental responses. This function might also be linked to reticulate mutant phenotypes (Beltran et al., 2018). These phenotypes are characterized by specific aberrations in the development of mesophyll cells, while vascular tissue seems to be unaffected although most genes affected in reticulate mutants are strongly expressed in this tissue (Lundquist et al., 2014). The reticulate phenotype can be explained by two models: the “supply” and the “signaling” hypothesis. The former suggests that primary metabolites are provided to the developing mesophyll tissue. If this supply is impaired, then mesophyll development is compromised. The signaling hypothesis proposes a molecular signal to be send out from the vascular tissue to control mesophyll development. Among others, amino acids might function both as supply and/or as a signal. This is consistent with the observation that administration of aromatic amino acids rescues the reticulate phenotype in the *cue1* mutant, in which the phosphoenolpyruvate/phosphate transporter 1 (PTT1) is affected (Lundquist et al., 2014). In addition, metabolite profiling of *re* and *rer3* reticulate mutants revealed lowered levels of aromatic as well as branched chain amino acids (Pérez-Pérez et al., 2013). Interestingly, the leaves of the IPMI SSU1 knockdown mutant shows phenotypic similarities to those of reticulate mutants, with green veins and pale interveinal regions as well as small narrow chloroplasts with reduced levels of starch. But the IPMI SSU1 knockdown mutant contains elevated levels of Leu, thus, the phenotype cannot be rescued by the administration of Leu (Imhof et al., 2014). However, the reticulate-like phenotype might suggest that reduced IPMI SSU1 function and its consequences on primary metabolism might interfere with sensory plastid function in the epidermis. Whether the IPMI SSU1 knockdown plants exhibit altered fitness under abiotic stress remains to be investigated, however, these plants already suffer from a strong pleiotropic phenotype under normal cultivation conditions (Imhof et al., 2014).

Beside the potential function in the rescue of the reticulate phenotype as described above, elevated levels of aromatic amino acids play a role in broad spectrum pathogen resistance by acting as precursors for phenylpropanoids, lignin, hormones and many other compounds (Lundquist et al., 2014; Tonnessen et al., 2015). Whether leucine or the other branched chain amino acids have similar functions is unclear at present.

The epidermal localization of IPMI SSU1 also applies to other green tissues like stems, siliques and maturing seeds strongly suggesting that this cell layer is the predominant tissue which hosts the Leu biosynthetic pathway, while major parts of the green tissue including the mesophyll, the main photosynthetic tissue, is excluded from biosynthesis of this amino acid.

Beside the epidermal localization, IPMI SSU1 was also detected in the root cortex and in cells associated with the phloem (Figure 6), which is in line with a role of this protein in early events of aliphatic glucosinolate biosynthesis (Imhof et al., 2014). Such a role is further supported by the detection of IPMI SSU1 in a narrow stripe potentially representing a single cell layer along the phloem-associated cells in flowering stalks expressing IPMI SSU2 and IPMI SSU3 (Supplementary Figure S5A). The weak fluorescence was found in structures highly similar to sensory plastids and no equivalent fluorescence was observed in non-transformed wild-type plants.

### IPMI SSU2 and IPMI SSU3 Are Expressed in Both Phloem-Associated and Xylem-Associated Cells

As expected from previous expression studies of genes involved in glucosinolate biosynthesis and regulation and from in tissue-specific proteomic studies (Reintanz et al., 2001; Chen et al., 2003; Grubb et al., 2004; Tantikanjana et al., 2004; Schuster et al., 2006; Gigolashvili et al., 2007, 2008; He et al., 2010; Li et al., 2011; Imhof et al., 2014; Svozil et al., 2015; Nintemann et al., 2018), both IPMI SSU2 and IPMI SSU3 were found to be expressed along vascular tissues. In green parts of adult plants particularly in flowering stalks, these proteins were found in chloroplasts of cells located in the periphery of the phloem (Figures 5D, 7A). However, we also found these proteins in parenchymatic cells situated proximal to the xylem. A similar pattern has been observed for the flavin monoxygenase FMO_GS–OX1_, which is involved in side chain modification of aliphatic glucosinolates, catalyzing one of the last steps in the biosynthesis of these metabolites (Li et al., 2011) as well as for the cytochrome P450 protein CYP83A1, required for core structure formation of aliphatic glucosinolates (Nintemann et al., 2018). Additionally, such a pattern was also seen when a nucleus-targeted 3xGFP reporter protein was expressed under the control of the *BCAT4* promoter (pBCAT4:3xGFP-NLS) (Supplementary Figures S5B,C). BCAT4 catalyzes the committed step of Met-derived glucosinolate biosynthesis (Schuster et al., 2006). Thus, in total six different proteins involved in glucosinolate biosynthesis are expressed not only in phloem-associated cells but also in parenchymatic cells proximal to the xylem demonstrating that these cells play a role in all phases of the biosynthesis of Met-derived aliphatic glucosinolates. It is presently unknown whether these cells exhibit any additional functional specification apart from their role in specialized metabolism. The observation that biosynthesis of these specialized compounds might also occur in the proximity to the xylem fits well with the transport of glucosinolates in the xylem sap (Andersen et al., 2013; Madsen et al., 2014).

By using transcriptional/translational fusions we found that the expression of BCAT4 and IPMI SSU2/IPMI SSU3 is restricted to cells located at the proximal boundary of the phloem and at the inwards oriented side of the xylem of flowering stalks (Figure 6 and Supplementary Figures 5B,C). The localization of the *BCAT4* promoter driven 3XGFP (pBCAT4:3XGFP-NLS) in the cells of the phloem periphery could potentially coincide with sulfur rich S-cells, which contain large amounts of glucosinolates. These cells are situated between the phloem parenchyma and the endodermis (Koroleva et al., 2000, 2010). However, it seems unlikely that BCAT4 and IPMI SSU2/IPMI SSU3 are expressed in these cells, since these sulfur-rich cells are biosynthetically inactive (Xu et al., 2019). This is supported by the observation that *BCAT4* promoter driven expression of GFP (pBCAT4:3XGFP-NLS) was seen in nuclei of relatively small and short cells with a morphology completely different from the extremely long S-cells (Supplementary Figures S5B,C). This is consistent with the conclusion drawn from the expression analysis of the above mentioned flavin monoxygenase FMO_GS–OX1_ and CYP83A1, which excludes expression of these proteins in S-cells (Li et al., 2011; Nintemann et al., 2018). As suggested by Nintemann et al. (2018) for CYP83A1, we favor the presence of BCAT4 and IPMI SSU2/IPMI SSU3 in phloem parenchyma cells. Nevertheless, cells synthesizing aliphatic glucosinolates and cells storing these compounds are in close vicinity to each other. Likewise, parenchymatic cells located proximal to the xylem express these proteins suggesting that Met chain elongation and probably the complete biosynthesis of aliphatic glucosinolates occurs in this tissue. These compounds could potentially be transferred into the xylem, which serves as transport system for glucosinolates (Andersen et al., 2013). Unlike the situation in the periphery of the phloem, all of the glucosinolates synthesized near the xylem are most likely transported into other plant parts since storage of glucosinolates has not been observed in the vicinity of the xylem (Koroleva et al., 2010).

### In Roots, IPMI SSU1 and IPMI SSU2/IPMI SSU3 Are Found in Tissues Surrounding the Central Cylinder

In young roots, expression of IPMI SSU2 and IPMI SSU3 was found in cells along the central cylinder, which is consistent with the biosynthesis of glucosinolates in this organ (Andersen et al., 2013). In canola, storage of glucosinolates has been demonstrated in the inner periderm of roots showing secondary growth whereas an accumulation to a far lesser extent seems to occur in phloem parenchyma of roots before secondary growth initiates (McCully et al., 2008). If a similar scenario is assumed for *Arabidopsis*, storage in the phloem parenchyma would be close to or even coincide with tissues where IPMI SSU2 and IPMI SSU3 are expressed (Figure 6). However, further detailed investigations of glucosinolate storage in roots of *Arabidopsis* are required to verify this assumption.

In roots, all three small IPMI subunits are found in colorless plastids. Those containing IPMI SSU1 have frequently been found to form stromules (Figure 6A, inset) (Köhler and Hanson, 2000). Although they are morphologically similar to each other, these plastids seem to be functionally specialized having different biological roles in plant metabolism, as suggested by the presence or absence of the different IPMI proteins and presumably also of other enzymes involved in Leu biosynthesis or Met chain elongation. The exclusion of IPMI SSU1 from large parts of the green tissue indicates that roots might also play an important role in Leu or even BCAA biosynthesis. The relatively strong expression of IPMI SSU2 and IPMI SSU3 supports a substantial role of roots in biosynthesis of Met-derived glucosinolates.

### Spatial Separation of IPMI SSU1 From IPMI SSU2 and IPMI SSU3

Considering the results of our studies, two features determine the functional specification of IPMI in Leu biosynthesis (primary metabolism) and Met chain elongation (specialized metabolism) in *Arabidopsis*. First, the different primary sequences within the SRR of the small subunits define the substrate preferences of the different heterodimers. Second, there seems to be an almost complete spatial separation of IPMI heterodimers containing either IPMI SSU1 or IPMI SSU2/IPMI SSU3 assigning distinct functions to certain cell and tissue types (Figure 9). IPMI SSU2 and IPMI SSU3 are apparently co-expressed in identical organelles, in almost identical cells and tissues, suggesting the heterodimers containing these proteins to have very similar or identical functions. However, two exceptions have been observed: in hypocotyls, IPMI SSU2 was found in further peripheral cells in comparison to IPMI SSU3 (Figures 3E,F,H,I), and in cotyledons, where IPMI SSU2 was seen in isolated epidermal cells (Figures 3A,C), a pattern which we never observed for IPMI SSU3. Identical expression patterns of IPMI SSU2 and IPMI SSU3 were found in many different plants from different generations, which makes it highly unlikely that the pattern are influenced by unknown regulatory loci segregating in the Col/Ws amalgam of the *ipmi ssu2-1/ipmi ssu3-1* nuclear background. In addition, for IPMI SSU1 we used two different constructs, in which the reading frame is fused to different reporter genes (IPMI SSU1:RFP/IPMI SSU2:ECFP). We obtained identical results with these different constructs also analyzed in plants from different generations.

The differential expression can be summarized as follows (Figure 9): In green parts of adult plants analyzed in this study, IPMI SSU1 was almost exclusively found in small plastids in epidermal cells. Interestingly, this suggests that extensive transport processes are required for a full supply of the whole plant with Leu. The small IPMI SSU1-containing plastids were also detectable in phloem associated cells in flowering stalks. With regard to size and tissue localization these plastids are identical to sensory plastids. In addition, plastids containing IPMI SSU1 exhibited weak, mostly undetectable autofluorescence typical for chlorophyll, which is consistent with the detection of proteins required for photosynthesis is these organelles (Supplementary Figure S3). Sensory plastids contain IPMI proteins as well as other enzymes required for Leu biosynthesis (Beltran et al., 2018). In roots, IPMI SSU1 was predominantly detected in small plastids of cortex cells surrounding the central cylinder and in roots hairs, which are part of the epidermal layer. High expression of this protein is also evident in root tips.

In above-ground parts, IPMI SSU2 and IPMI SSU3 were found in chlorophyll-containing chloroplasts of cells in the periphery of the phloem and in parenchymatic cells located proximal to the xylem (Figures 5D, 7A). A very similar (or almost identical) tissue localization was found BCAT4, also active in Met chain elongation (Supplementary Figures 5B,C) (Schuster et al., 2006). Neither IPMI SSU2 and IPMI SSU3 nor BCAT4 were expressed in S-cells, the major storage sites of glucosinolates in the flowering stalk and leaves. In roots, IPMI SSU2 and IPMI SSU3 were seen in cells surrounding the central cylinder, where they seem to be located proximal to those cells expressing IPMI SSU1.

IPMI SSU2 and IPMI SSU3 are physically separated from IPMI SSU1 at the subcellular level. This might also hold true for the cellular level, but in case of the weak expression observed along the phloem we were not able to assign the fluorescing plastid-like structures to specific cells. Nevertheless, our studies indicate a spatial separation of Met chain elongation pathway and Leu biosynthesis. A previous study suggested an additional role of IPMI SSU1 in early phases (at least the first cycle) of Met chain elongation suggested by the accumulation of intermediates of Met chain elongation (mainly C3, less C4) and the biosynthesis of predominantly short-chain glucosinolates (C3 and C4) in the *ipmi ssu2-1/ipmi ssu3-1* double knockout mutant (Imhof et al., 2014). The involvement of IPMI SSU1 in glucosinolate biosynthesis would require intercellular transport processes of intermediates between plastids in different cells. This appears to be straightforward in the phloem, where cells expressing IPMI SSU1 were in close proximity to cells expressing IPMI SSU2 and IPMI SSU3 (Supplementary Figure S5A). However, the IPMI SSU1 knockdown plants show a strong pleiotropic phenotype including elevated levels of jasmonic acid (and derivatives of it). The increase of these hormones promotes glucosinolate biosynthesis and might thus lead to an “artificial” excessive accumulation of short-chain intermediates. As argued in Imhof et al. (2014), the accumulation of short-chain glucosinolates in the *ipmi ssu2-1/ipmi ssu3-1* double knockout mutant also demonstrates that IPMI SSU1 can compensate for the absence of IPMI SSU2 and -3 in the biosynthesis of these metabolites. To which extent this participation occurs in wild-type plants with three intact IPMI SSU genes, cannot be finally assessed.

We have not analyzed all tissues in all developmental stages and in some tissues there might be expression beyond detection levels. Thus, we cannot exclude additional expression patterns to the above described expression patterns. However, our study provides clear evidence that in *Arabidopsis* the SRR determines the substrate preference of the different heterodimeric enzyme Isopropylmalate isomerases and that this functional specialization is underpinned by different spatial localization of the distinct small subunits. Both features guarantee clear functions of the different heterodimers in either Leu biosynthesis or Met chain elongation albeit the large subunit participates in both pathways.

## Data Availability Statement

All datasets generated for this study are included in the article/Supplementary Material.

## Author Contributions

KL, JI, and SB planned and designed the work. KL, KC, JI, and CC performed the experiments and measurements, and analyzed the data. KL, KC AS, and CC carried out and assisted the microscopy and image analysis. KL, JI, KC, AS, CC, BH, and SB contributed to the interpretation of results. SB wrote the manuscript. All the authors made intellectual contributions to the study, read the manuscript, and approved the submission.

## Conflict of Interest

The authors declare that the research was conducted in the absence of any commercial or financial relationships that could be construed as a potential conflict of interest.

## Footnotes

1 http://www.4base-lab.de/index_4.html

2 https://www.lgcgroup.com/

3 https://www.ebi.ac.uk/Tools/msa/clustalw2/
