# Supplementary material for: In Arabidopsis thaliana Substrate Recognition and Tissue- as Well as Plastid Type-Specific Expression Define the Roles of Distinct Small Subunits of Isopropylmalate Isomerase
Source: Front Plant Sci. 2020 Jun 16;11:808. doi: 10.3389/fpls.2020.00808 (PMC7308503; doi:10.3389/fpls.2020.00808)
Supplement: Supplementary file 1 [file Data_Sheet_1.PDF]

```
AtIPMISSU2      MA-----------YSLPTFPQALPCSSTKTSSSLATFRSPFLRFNGSTSLIPS-SISITS    48
AtIPMISSU1      MAAS-------LQSANPTLSRTL--ASPNKPSSFATFRSPFLRFNSTSVASNF-K----P    46
AtIPMISSU3      MATS-------QQFLNPTLFKSL--ASSNKNS-CTLCPSPFLQLKSASTIFNY-K----P    45
OsIPMISSU       MAAAAAAPALSLAEAAPVTAV------------LAPCPTPSRTFRRRSWVAAICRPALKC    48
MtLeuD          ------------------------------------------------------------    0
SmLeuD          ------------------------------------------------------------    0
MjLeuD          ------------------------------------------------------------    0
MjHACN          ------------------------------------------------------------    0


AtIPMISSU2      RGTSSPTIIPRAAASESDSNEALANTTFHG-LCYVLKDNIDTDQIIPAGAACTFPSNQQE    107
AtIPMISSU1      LVSREASSSF----VTRSAAEPQERKTFHG-LCYVVGDNIDTDQIIPAEFLTLVPSNPEE    101
AtIPMISSU3      LTSSSATIITRVAASSSDSGESITRETFHG-LCFVLKDNIDTDQIIPAEYGTLIPSIPED    104
OsIPMISSU       HHSRPLTAVAAAAAAAAAAGDSTSAGVFHG-ECFVVGDNIDTDQIIPAEHLTLVPSKPDE    107
MtLeuD          ---------------------MEAFHTHSGIGVPLRRSNVDTDQIIPAVFLKRVT-----    34
SmLeuD          ---------------------MEEFTIYTGTTVPLMNDNIDTDQILPKQFLKLID-----    34
MjLeuD          --------------------------MIKG-RVWKFGNNVDTDAILPARYLVYT-----K    28
MjHACN          -------------------------MIIKG-RAHKFGDDVDTDAIIPGPYLRTT-----D    29
                                             *       .::*** *:*

AtIPMISSU2      RDEIAAHALSGLP---D-F-HKTRFIEPGENRSKYSIIIGGENFGCGSSREHAPVCLGAA    162
AtIPMISSU1      YEKLGSYALVGLP---A-S-YKERFVQPGEMKTKYSIIIGGENFGCGSSREHAPVCLGAA    156
AtIPMISSU3      REKLGSFALNGLP---K-F-YNERFVVPGEMKSKYSVIIGGDNFGCGSSREHAPVCLGAA    159
OsIPMISSU       YRKLGSFAFVGLP---TAA-YPTPFVAPGEETTRYAVIIGGANFGCGSSREHAPVALGAA    163
MtLeuD          RTGFEDGLFAGWR-------SDPAFVLNLSPFDRGSVLVAGPDFGTGSSREHAVWALMDY    87
SmLeuD          KKGFGKYLMYEWRYLDNNYTENPDFIFNQPEYREASILITGDNFGAGSSREHAAWALADY    94
MjLeuD          PEELAQFVMTGAD---PDF-PK--KVKP------GDIIVGGKNFGCGSSREHAPLGLKGA    76
MjHACN          PYELASHCMAGID---ENF-PK--KVKE------GDVIVAGENFGCGSSREQAVIAIKYC    77
                   :    :                :          ::: * :** *****:*   :

AtIPMISSU2      GAKAIVAESYARIFFRNSVATGEVFPLES-EV----RVCEECKTGDTVTIELSDS-GGLL    216
AtIPMISSU1      GAKAVVAQSYARIFFRNSVATGEVYPLDS-EV----RVCDECTTGDVATVELR-EGDSIL    210
AtIPMISSU3      GAKAVVAESYARIFFRNCVATGEIFPLES-EV----RICDECKTGDVVTIEHKEDGSSLL    214
OsIPMISSU       GARAVVAEGYARIFFRNSVATGEVYPLELADT----GAWKECKTGDVVTVELD---NCVM    216
MtLeuD          GFRVVISSRFGDIFRGNAGKAGLLAAEVAQDDVELLWKLIEQSPGLEITANLQDR---II    144
SmLeuD          GFKVIVAGSFGDIHYNNDLNNGILPIIQPKEV---RDKLAKLKPTDEVTVNLFEQ---KI    148
MjLeuD          GISCVIAESFARIFYRNAINVGLPLIE---CK----GISEKVNEGDELEVNLETG---EI    126
MjHACN          GIKAVIAKSFARIFYRNAINVGLIPII---A------NTDEIKDGDIVEIDLDKE---EI    125
                *   :::  :. *.  *    *                  : .       :        :

AtIPMISSU2      TNHTTGKNYKL-KSIGDAGPVID-AGGIFAYARMMGMIPSLA---------------       256
AtIPMISSU1      INHTTGKEYKL-KPIGDAGPVID-AGGIFAYARKAGMIPSAAA--------------       251
AtIPMISSU3      INHTTRKEYKL-KPLGDAGPVID-AGGIFAYARKAGMIPSA----------------       253
OsIPMISSU       INHTSGKQYKL-KPIGDAGPVIE-AGGIFAYARKTGMIASKSA--------------       257
MtLeuD          TAATVVLPFKIDDH--SAWRLLEGLDDIALTLRKLDEIEAFEG-ACAYWKPRTLPAP       198
SmLeuD          YSPVGDFSFDIDGE--WKHKLLNGLDDIGITLQYEDLIAQYEQNRPSYWH-------       196
MjLeuD          KNLTTGEVLKGQKLPEFMMEILE-AGGLMPYLKKKMAESQ-----------------       165
MjHACN          VITNKNKTIKCETPKGLEREILA-AGGLVNYLKKRKLIQSKKGVKT-----------       170
                         .          ::   ..:    :
```

Supplementary Figure. S1. Alignment of IPMI SSU (LEUD) from various pro- and eukaryotic organisms. The following sequences are aligned: AtIPMI SSU1 (At2g43090), AtIPMI SSU2 (At2g43100) and AtIPMI SSU3 (At3g58990)from *Arabidopsis thaliana*, OsIPMI SSU (AAQ67235.1) from *Oryza sativa*, MtLeuD (WP_003914930) from *Mycobacterium tuberculosis*, LeuD and HACN (MjLeuD WP_064496959 from MjHACN WP_010870784) *Methanocaldococcus jannaschii* and SmLeuD (WP_002262705) from *Streptococcus mutans.* The substrate recognition region (SRR) is highlighted in yellow. An *asterisk indicates positions which have fully conserved residue (highlighted in red). A : (colon) indicates conservation between groups of strongly similar properties. A . (period) indicates conservation between groups of weakly similar properties. Alignment as generated with CLUSTAL OMEGA (1.2.4) multiple sequence alignment (https://www.ebi.ac.uk/Tools/msa/clustalo/).
