# Supplementary material for: In Arabidopsis thaliana Substrate Recognition and Tissue- as Well as Plastid Type-Specific Expression Define the Roles of Distinct Small Subunits of Isopropylmalate Isomerase
Source: Front Plant Sci. 2020 Jun 16;11:808. doi: 10.3389/fpls.2020.00808 (PMC7308503; doi:10.3389/fpls.2020.00808)
Supplement: Supplementary file 2 [file Data_Sheet_2.PDF]

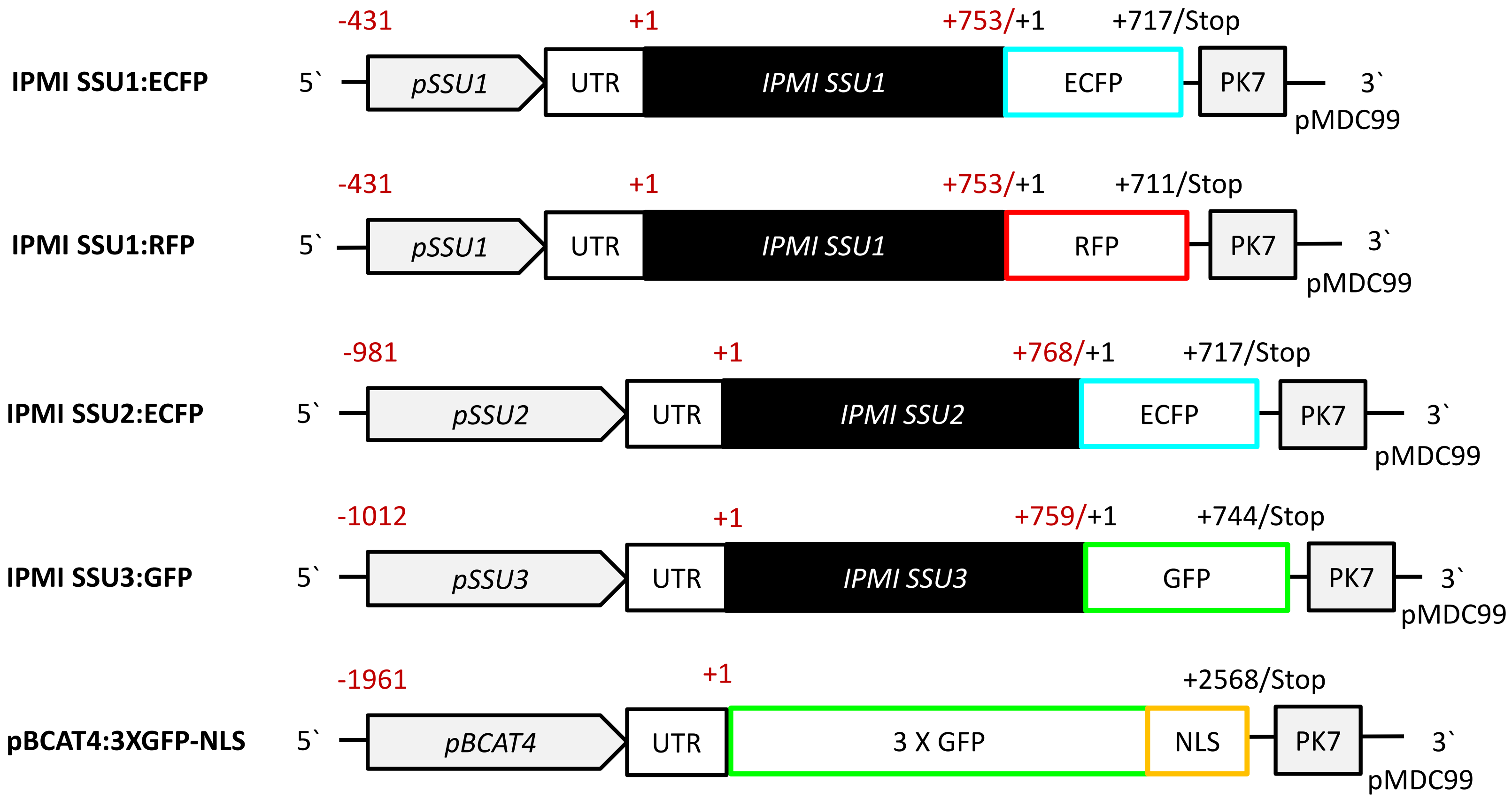

Supplementary Figure. S2. Reporter constructs used for expression analysis. Numbering is given with respect to the NATG (A = +1, N = -1). Numbers given in red delimit regions investigated, numbers given in black belong to the reading frames of the various fluorescent proteins.
