# Supplementary material for: In Arabidopsis thaliana Substrate Recognition and Tissue- as Well as Plastid Type-Specific Expression Define the Roles of Distinct Small Subunits of Isopropylmalate Isomerase
Source: Front Plant Sci. 2020 Jun 16;11:808. doi: 10.3389/fpls.2020.00808 (PMC7308503; doi:10.3389/fpls.2020.00808)
Supplement: Supplementary file 3 [file Data_Sheet_3.PDF]

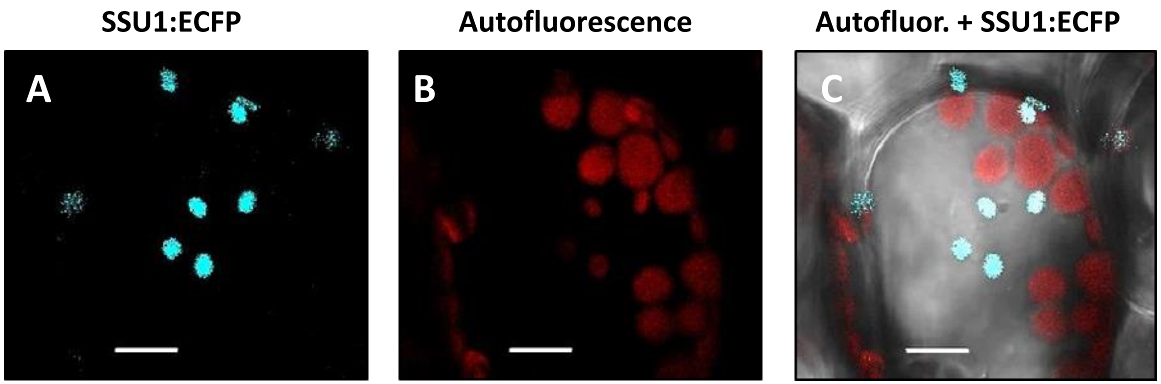

Supplementary Figure S3. (A-C) IPMI SSU1:ECFP-positive plastids (**(A)** cyan) and chloroplasts (**(B)** red) located in different cell layers. **(C)** Overlay of **(A)** and **(B)**. The lower mesophyll cell (MC) contains large chloroplasts (longitudinal diameter about 5 to 8 µm), while the epidermal cells contain small plastids (longitudinal diameter about 4 µm). In most cases no autofluorescence was detected in these smaller plastids, but in some instances typical chlorophyll autofluorescence was detected as shown in **(B)**. Scale bars: 10 µm **(A-C)**.
