# Supplementary material for: In Arabidopsis thaliana Substrate Recognition and Tissue- as Well as Plastid Type-Specific Expression Define the Roles of Distinct Small Subunits of Isopropylmalate Isomerase
Source: Front Plant Sci. 2020 Jun 16;11:808. doi: 10.3389/fpls.2020.00808 (PMC7308503; doi:10.3389/fpls.2020.00808)
Supplement: Supplementary file 4 [file Data_Sheet_4.PDF]

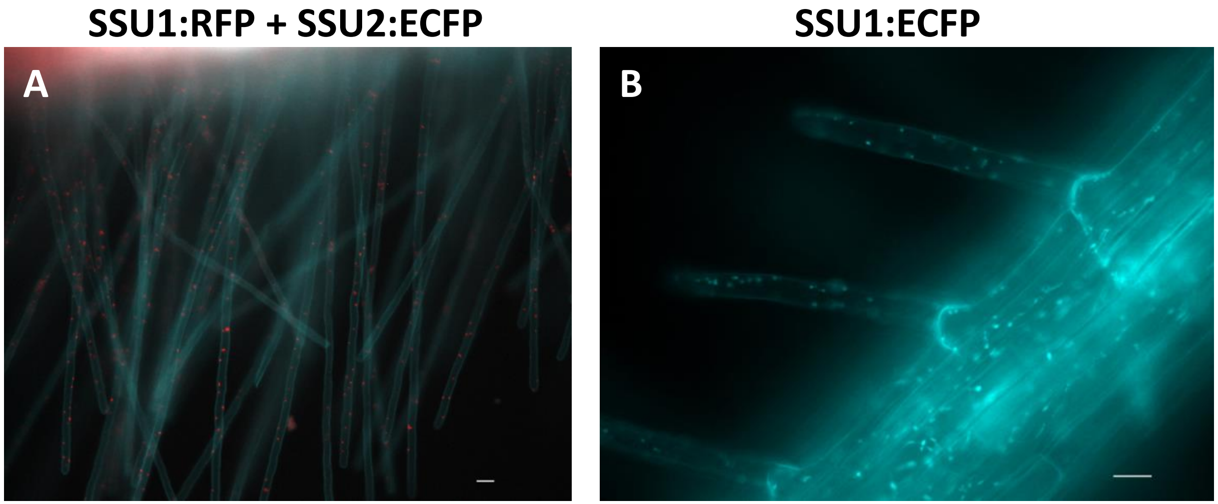

Supplementary Figure S4. IPMI SSU1:RFP **(A)** and IPMI SSU1:ECFP-positive plastids **(B)** are seen in root hairs of 14-days-old Arabidopsis plants. Scale bars 20 µm.
