# Supplementary material for: In Arabidopsis thaliana Substrate Recognition and Tissue- as Well as Plastid Type-Specific Expression Define the Roles of Distinct Small Subunits of Isopropylmalate Isomerase
Source: Front Plant Sci. 2020 Jun 16;11:808. doi: 10.3389/fpls.2020.00808 (PMC7308503; doi:10.3389/fpls.2020.00808)
Supplement: Supplementary file 5 [file Data_Sheet_5.PDF]

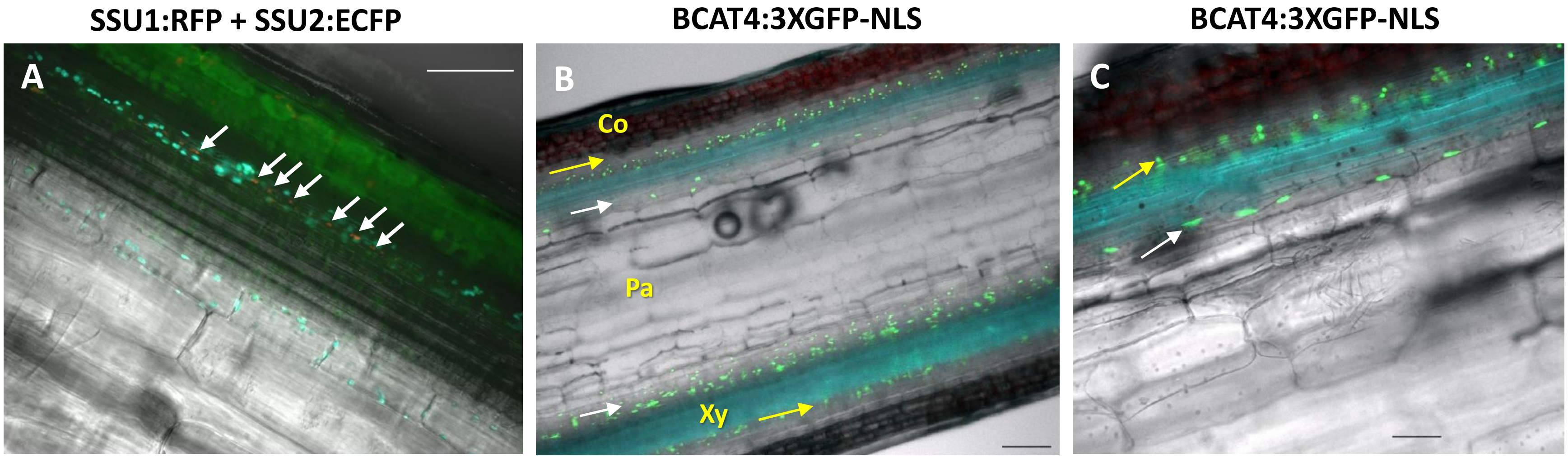

Supplementary Figure. S5. Longitudinal section of flowering stalk. **(A)** In dual reporter line, weak red fluorescence of IPMI SSU1:RFP (red, highlighted by white arrows) was seen in a narrow stripe along with cells expressing IPMI SSU2:ECFP (cyan). These cells might represent phloem parenchyma cells. **(B, C)** A longitudinal section exhibits BCAT4 promoter activity in cells in close proximity to the phloem (yellow arrow) and xylem (white arrow). The extreme length of S-cells (i.e. glucosinlate storing, Sulphur rich cells) does not fit the cell type indicated by numerous fluorescing nuclei. Remarkably, many nuclei indicating BCAT4 expression in xylem associated cells exhibit longish shapes whereas the nuclei of those cells associated with the phloem seem to have predominantly globular shapes. Co: cortex; Pa: parenchyma cells; Xy: xylem. Scale bars: **(A, C)** 50 µm; **(B)** 100 µm.
