# Supplementary material for: In Arabidopsis thaliana Substrate Recognition and Tissue- as Well as Plastid Type-Specific Expression Define the Roles of Distinct Small Subunits of Isopropylmalate Isomerase
Source: Front Plant Sci. 2020 Jun 16;11:808. doi: 10.3389/fpls.2020.00808 (PMC7308503; doi:10.3389/fpls.2020.00808)
Supplement: Supplementary file 6 [file Presentation_1.pdf]

Supplementary Method S1: Standard TAIL-PCR program

primer	temperature [°C]	time [sec]	cycles
pMDC99-TR1 + Degenerated-Primer.1	94	60	1
	94	30	5
	59	60	
	72	150	
	94	30	1
	25	180	
	gradient 25 → 70	180	
	72	150	
	94	30	15
	65	60	
	72	150	
	94	30	
	65	60	
	72	150	
	94	30	
	40	60	
	72	150	
	72	300	1
pMDC99-TR2 + Degenerated-Primer.1	94	60	1
	94	30	12
	65	60	
	72	150	
	94	30	
	65	60	
	72	150	
	94	30	
	40	60	
	72	150	
	72	300	1
pMDC99-TR3 + Degenerated-Primer.1	94	60	1
	94	45	20
	40	60	
	72	150	
	72	300	1
