# Supplementary material for: In Arabidopsis thaliana Substrate Recognition and Tissue- as Well as Plastid Type-Specific Expression Define the Roles of Distinct Small Subunits of Isopropylmalate Isomerase
Source: Front Plant Sci. 2020 Jun 16;11:808. doi: 10.3389/fpls.2020.00808 (PMC7308503; doi:10.3389/fpls.2020.00808)
Supplement: Supplementary file 7 [file Table_1.pdf]

Supplementary Table S1: Localization of T-DNA insertions in homozygous IPMI SSU1:RFP, IPMI SSU2:ECFP und IPMI SSU3:GFP lines.

Line	**SSU1:RFP #5**	**SSU2:ECFP #4**	**SSU3:GFP #1**
Gene	At2g43090	At2g43100	At3g58990
Size of the IPMI SSU gene	-431/+753 (1184 bp)	-981/+768 (1749 bp)	-1012/+759 (1771 bp)
Size of the FP gene	711 bp	716 bp	743 bp
T-DNA insertion site	double insertion in non-encoding region between At2g35743 and At2g35744<br>Chr. 2 pos. 15029138	single insertion in non-encoding region between At5g19690 and At5g19700<br>Chr. 5 pos. 6658855	single insertion<br>-22 nt upstream of *ATG* of At1g76490<br>Chr. 1 pos. 28695493

Supplementary Table S2. Oligonucleotide sequences

Name	Sequence (5' to 3')
ssu3/1OE.R	gctgattgcagagaagccgccattgttctgctacttcttaagtgaagcagg
ssu3/1OE.H	cctgcttcacttaagaagtagcagaacaatggcggcttctctgcaatcagc
ssu3/1seq.H	cgtttaggacttagaatggcg
ssu1/3OE.R	gggtttaaaaattgctgagaagtcgccattgtttgtttctctttgctgtgg
ssu1/3OE.H	ccacagcaaagagaaacaaacaatggcgacttctcagcaatttttaaaccc
ssu1comp.H	atgcagtcgagcggcgcgcccgcagttctcttctggagc
ssu1comp.R	gacttcgagtttaattaagccattcagaattccaaacg
ssu2comp.H	atgcagtcgagcggcgcgccgctggaccagtgattgatgc
ssu2comp.R	gacttcgagtttaattaaggcgttataaagtaggagtcg
ssu3comp.H	atgcagtcgagcggcgcgccgcaagtggaaggaaatacg
ssu3comp.R	gacttcgagtttaattaagatctcatttggtagattgc
ssu3/1FOE.R2	attcctctggattcgaagggatgagagtgtgccgtactccgcgggaatgatttg
ssu1FOE.H	ccttcgaatccagaggaat
ssu1/3FOE.R	atcttctggaatcgaagggacgagagtgagaaactcggcggggattatttg
ssu3FOE.H	ccttcgattccagaagatcgc
LSUPro.H	atgctgctgcagggcttcgtggagctcatcc
LSUPro.R	atgctgctgcaggccattgattcttcttcgtcttc
LSUPro.H2	atgctgggatccggcttcgtggagctcatcc
LSUPro.R2	atgctgggatccgccattgattcttcttcgtcttc
LSUPro.H3	atgctgcccgggggcttcgtggagctcatcc
LSUProseq1	ggtttttacatggacctcaacc
LSUProseq2	aacttaggaaaatcatagagttaagc
LSUProseq3	atagtcccaaatagcaagcaac
LSU-Chlp.target	atgcaggaattcgaaaactgggatgacaa

ssu1 -Chlp.target	tacgatcatatggcatcctcatcgttcgtca
ssu1.R-AatII	tacgatgacgtcagcagcagcagatggaatc
ssu2 -Chlp.target	tacgatcatatgtcctccccgaccatcatc
ssu2.R-AatII	tacgatgacgtcagctaatgatggaatcattccc
ssu3 -Chlp.target	tacgatcatatgtccgccacgatcatcac
ssu3R.AatII	tacgatgacgtcagcagaaggaatcatgccg
LSU.R-NotI	atgcaggcggccgcgcctactgcaagaactcccttg
pMDC99-TR1	ccgatggctgtgtagaagtactc
pMDC99-TR2	agtactcgccgatagtggaaacc
pMDC99-TR3	cgacaagctcgagtttctcc
At2g35740.H	ggttttaatcggttctgatactactacc
At2g35740.R	gtcctaactgtagctgtgggtg
At5g19690.H	tttgtcgggtggaagacgtc
At5g19690.R	tggagtcatgtccagaagacac
At1g76490.H	cgtgataagtgtgaaggagag
At1g76490.R	tacgaccattggtctttgcg
At1g76490-2	aagcatcaccagaggcacg
Degenerated-Primer.1	(a/t)gtgnag(a/t)ancanaga
pSAT6seq.up	cagtgccacccataatacccat
CFP.seq	aacttgtggccgtttacg
LB-XL	gtggaccgcttgctgcaac
FLAG-LB1	cactgggattcgtcttggacaac
GUSBACK	cttgtaacgcgctttcccacc
pSAT6.H-(BamHI)	atgcagggatcctgatggtgagcaagggcgag
SSU1-ECFP.H(PacI)	gacttcgagtttaattaacatggaggatgtgaagttctgtctgg
SSU1-ECFP.R(SgsI)	atgcagtcgagcggcgcgccaagcagcagcagatggaatcattcc
SSU2-ECFP.H(PacI)	gacttcgagtttaattaagctgcttgattttgacttcagg
SSU2-ECFP.R(SgsI)2	atgcagtcgagcggcgcgccaagctaatgatggaatcattccc
SSU3-GFP.H	atgcagggatccttaaatcgctggttcgcgaag
SSU3-GFP.R	atgcagcccgggagcagaaggaatcatgccg
